# A quantitative model of nitrogen fixation in the presence of ammonium

**DOI:** 10.1371/journal.pone.0208282

**Published:** 2018-11-29

**Authors:** Keisuke Inomura, Jason Bragg, Lasse Riemann, Michael J. Follows

**Affiliations:** 1 Department of Earth, Atmospheric and Planetary Sciences, Massachusetts Institute of Technology, Cambridge, MA, United States of America; 2 National Herbarium of New South Wales, The Royal Botanic Gardens and Domain Trust, Sydney, NSW, Australia; 3 Marine Biological Section, Department of Biology, University of Copenhagen, Helsingør, Denmark; Universite Paris-Sud, FRANCE

## Abstract

Nitrogen fixation provides bioavailable nitrogen, supporting global ecosystems and influencing global cycles of other elements. It provides an additional source of nitrogen to organisms at a cost of lower growth efficiency, largely due to respiratory control of intra-cellular oxygen. Nitrogen-fixing bacteria can, however, utilize both dinitrogen gas and fixed nitrogen, decreasing energetic costs. Here we present an idealized metabolic model of the heterotrophic nitrogen fixer *Azotobacter vinelandii* which, constrained by laboratory data, provides quantitative predictions for conditions under which the organism uses either ammonium or nitrogen fixation, or both, as a function of the relative supply rates of carbohydrate, fixed nitrogen as well as the ambient oxygen concentration. The model reveals that the organism respires carbohydrate in excess of energetic requirements even when nitrogen fixation is inhibited and respiratory protection is not essential. The use of multiple nitrogen source expands the potential niche and range for nitrogen fixation. The model provides a quantitative framework which can be employed in ecosystem and biogeochemistry models.

## Introduction

Nitrogen fixation, the conversion of dinitrogen gas to bioavailable nitrogen, is carried out by diverse prokaryotes termed diazotrophs. It plays a fundamental role in maintaining the productivity of aquatic and terrestrial environments both locally and globally. The development of quantitative, mechanistic models with which to interpret and predict the biogeography and rate of nitrogen fixation and their relationship to environmental factors is a significant challenge for carbon cycle and climate modeling [1–4].

### Diazotrophy in the presence of fixed nitrogen

The ability to fix nitrogen provides a clear ecological advantage in oligotrophic environments where nitrogen is limiting. This is at a cost of lower growth efficiency than an organism using fixed nitrogen [5] due to the direct cost of reducing N_2_ as well as the indirect cost of managing intra-cellular oxygen, which de-activates nitrogenase [6–10]. Strategies to manage oxygen include enhanced respiration [7,11–13], specialized heterocyst cells, and (in photo-autotrophic diazotrophs) temporal separation of nitrogen and carbon fixation [14–17]. The presence of reduced nitrogen also impacts nitrogen fixation. On the one hand, ammonium or nitrate offer a “cheaper” source of nitrogen for an organism [18] and the presence of fixed nitrogen can inhibit nitrogen fixation [19,20]. On the other hand, there is significant evidence showing that diazotrophs can and do often use both fixed nitrogen and dinitrogen [21].

Uptake of ammonium reduces the cell’s electron potential, which may inhibit the flow of reducing equivalents to nitrogenase [19]. High intracellular concentrations of ammonium have been observed to cause downregulation of *nif* genes and decrease synthesis of the nitrogenase complex [20], presumably because preferential consumption of fixed nitrogen leads to a more efficient growth. Despite this, active nitrogen fixation is observed in pelagic, mesopelagic and benthic marine environments where fixed nitrogen is present [21–23] and in marine waters, shelf-sediments and salt marshes where it is abundant [21,24–30]. In an extensive review [21], compiled evidence from diverse marine environments showing that, while sustained high concentrations of fixed nitrogen lead to the suppression of N_2_ fixation, it does not always exclude it. In benthic environments, nitrogen fixation occurs at significant rates at fixed-nitrogen concentrations exceeding 100 μM [31]. Nitrogen fixing organisms do utilize fixed nitrogen sources. For example, *Trichodesmium*, a marine, phototrophic diazotroph has been shown to assimilate nitrate in laboratory studies [21,32,33] and heterotrohic diazotrophs are seen to switch between, or simultaneously use, both dinitrogen and ammonium [32,34,35].

### Significance for ecological and biogeochemical models

Major current models of nitrogen fixation in the marine environment assumes that diazotrophs never utilize fixed nitrogen [36–39]. The ability to use fixed nitrogen extends the viable range for nitrogen fixation beyond highly nitrogen starved environments. It is observed to occur in nature, related to the availability of other substrates including phosphorus and organic carbon [21]. In order to appropriately capture the flexible nitrogen use of diazotrophs in ecological and biogeochemical models, enabling accurate evaluations of the biogeography and integrated rates of nitrogen fixation, we need a framework to predict when it is advantageous to a cell to fix nitrogen, use fixed nitrogen, or a combination of both.

As a step towards developing a quantitative, prognostic framework for modeling nitrogen fixation in diverse environments, here we bring together a quantitative model of the nitrogen fixing bacterium *Azotobacter vinelandii* [40] and published data from laboratory studies [34,41] in which it was grown in continuous culture with a sucrose and ammonium-based medium. We choose to develop the model around this system because *Azotobacter vinelandii* is a well-studied model organism (studied over a century [42]) and because the laboratory studies [34,41] are sufficient to quantitatively test and constrain a dynamic model of an organism using both N_2_ and ammonium under a variety of environmental conditions.

### Key findings from laboratory studies

**Fig 1:**
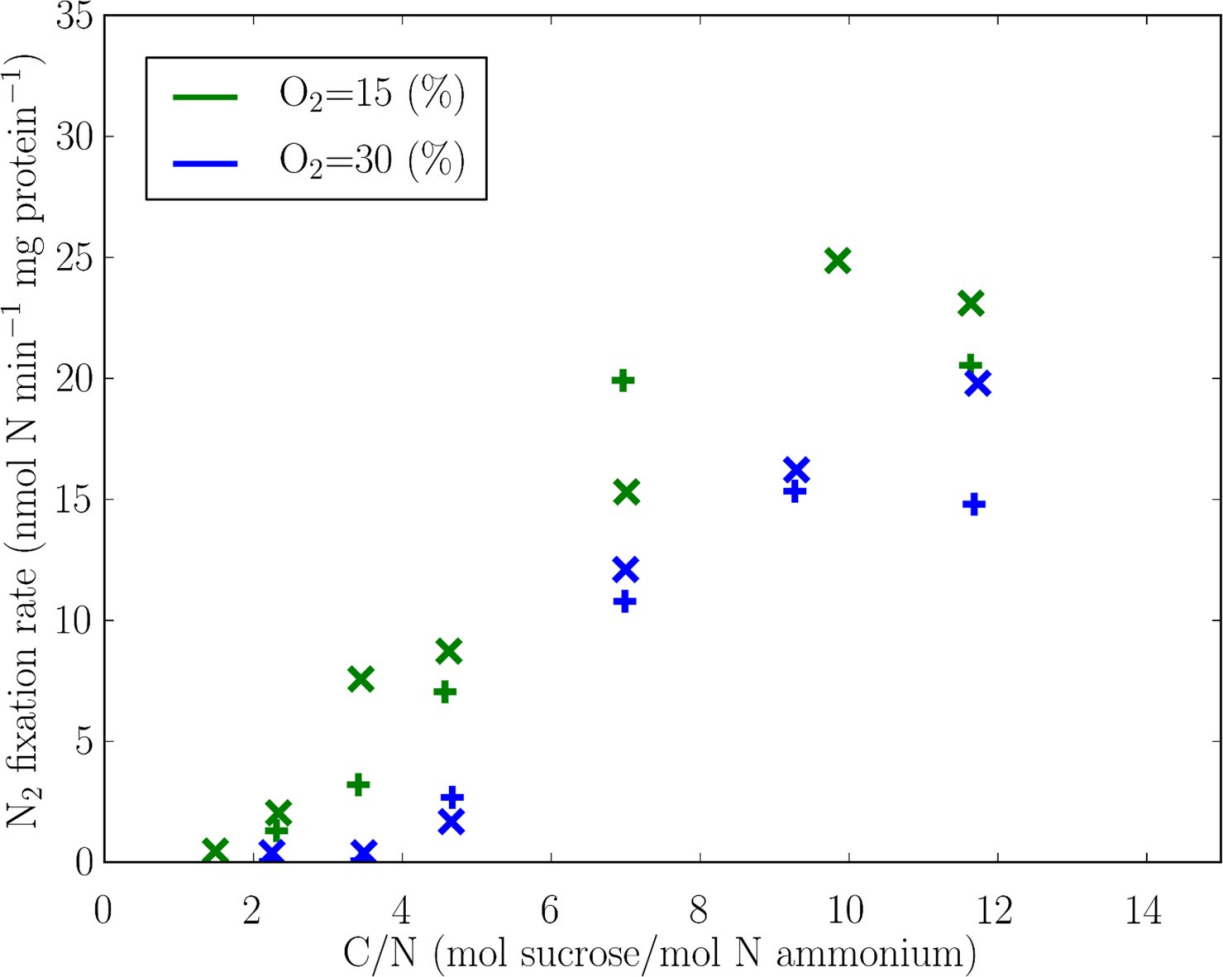
Nitrogen fixation rate per unit protein for various C/N (sucrose/ammonium) supply ratios and two oxygen concentrations in a continuous culture of *Azotobacter vinelandii*. Dilution rate is 0.15 (h^-1^) with constant ammonium supply of 0.375 mol m^-3^ h^-1^. Here 100% O_2_ equals 225 μM thus approximately O_2_ saturation under normal air composition at 30°C. Points are data redrawn from [34]; “+” based on acetylene reduction and “×”based on the rate of total nitrogen incorporation [34].

In the laboratory, the expression and rate of nitrogen fixation by the heterotrophic diazotroph *Azotobacter vinelandii* were examined as a function of relative rates of supply of organic carbon and fixed nitrogen [34]. They quantified this by the molar ratio of sucrose to ammonium in the incoming medium, C/N, where C/N = 1 is equivalent to the elemental ratio C:N = 12 (Table 1). Below a threshold C/N, nitrogen fixation was absent. Above the threshold, nitrogen fixation occurred at a rate which increased with C/N and eventually saturated (Fig 1). The threshold C/N increased and the nitrogen fixation rate decreased as the oxygen concentration increased [34,41]. Below the threshold, the organism’s sole nitrogen source was ammonium. At higher C/N, carbon in excess of the requirements to assimilate the NH_4_^+^ was used to deplete intra-cellular oxygen and fuel nitrogen fixation.

**Table 1:** Nomenclature of the used symbols in this main text. They are listed roughly in the order of appearance.

Symbol	Definition	Unit
C/N	Sucrose to ammonium ratio	mol sucrose mol N^-1^
C:N	Carbohydrate (in carbon) to ammonium ratio; C:N = 12C/N	mol C mol N^-1^
$R_{C/N}^{f}$	Lowest *R*_*C/N*_ at which nitrogen fixation is detected	mol sucrose mol N^-1^
*f*_*N2*_	The ratio of nitrogen fixation to the entire nitrogen source	dimensionless
$R_{C:N}^{f}$	$R_{C/N}^{f}$ in mol C mol N^-1^: $R_{C:N}^{f}$ = 12 $R_{C/N}^{f}$	mol C mol N^-1^
*ε*_*m*_	Diffusivity coefficient of the cell membrane layers	dimensionless
*ε*	Energy transfer efficiency	dimensionless

The aim of this study is to develop and apply a framework for interpreting and quantifying the cost and rates of nitrogen fixation in the presence of fixed nitrogen. In the Methods section we describe the modeling framework; further details are given in the supplementary material. In the Results section we simulate and interpret the nitrogen assimilation strategy of the heterotrophic diazotroph *Azotobacter vinelandii* under a variety of environmental conditions. In the Discussion, we discuss the implications for the conditions under which heterotrophic nitrogen fixation may be viable in real world environments.

## Methods

### Idealized metabolic model

**Fig 2:**
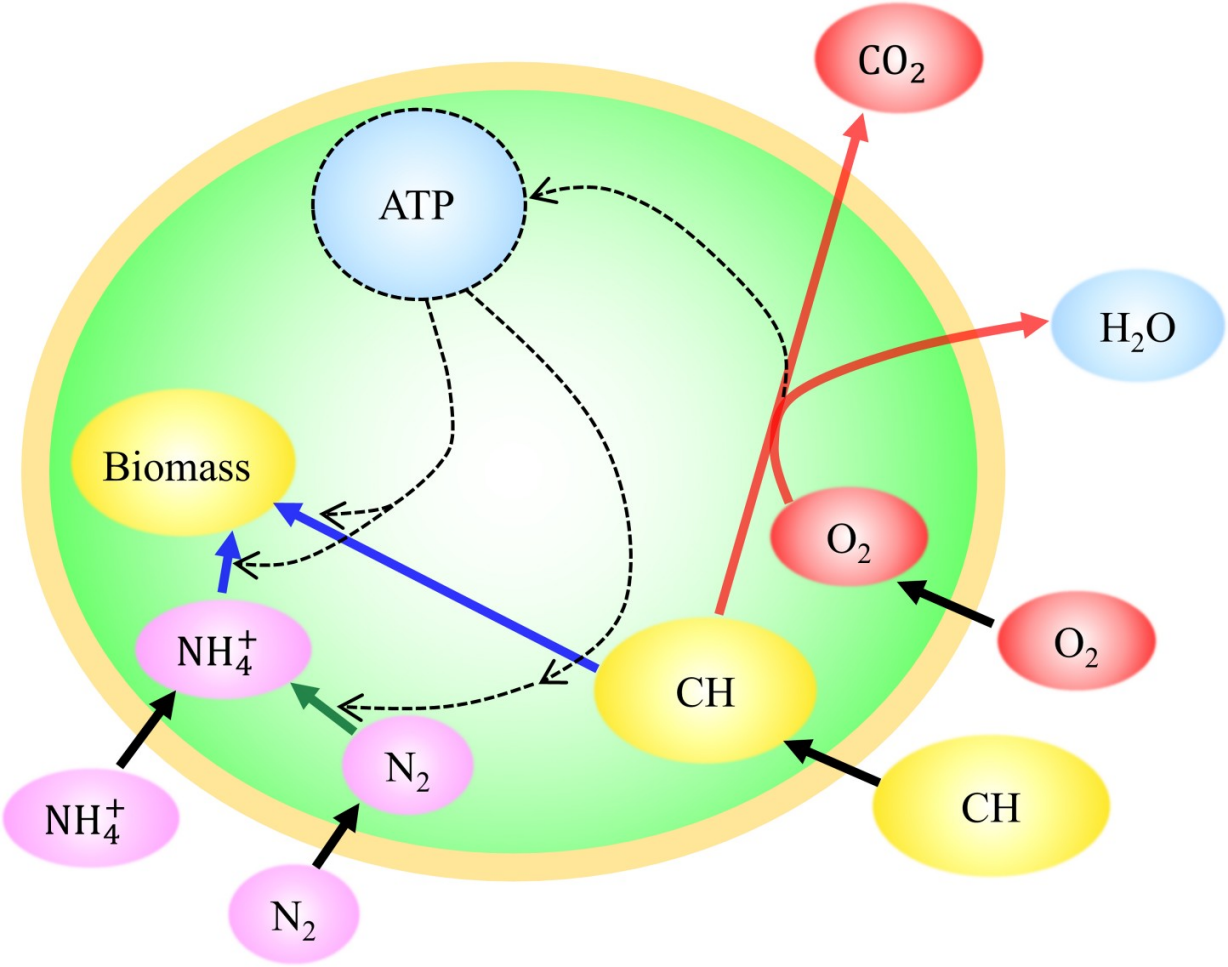
The cell flux model of a nitrogen fixing cell with ammonium uptake included. Solid black arrows, nutrient uptake; dashed black arrows, energy flow; red arrows, respiration; blue arrows, biomass production; green arrow, nitrogen fixation. CH represents carbohydrate. The competing nitrogen sources are computed based on maximizing biomass concentration.

The framework for this study is a previously published, simplified metabolic model of the heterotrophic soil bacterium *Azotobacter vinelandii* [40]. The model is depicted schematically in Fig 2 (and S1 Fig for detailed carbon fluxes), full details are provided in the Supplementary Material (S1 Text), and the code is available in Zenodo at https://zenodo.org/record/846300 (doi: 10.5281/zenodo.846300). Here we provide a brief description of the key elements of the model of [40] and the extensions developed for this study.

The framework consists of a simplified biochemical network to represent cellular metabolism constrained by mass, energy and electron balance at steady state [18]. The previously published model coupled diffusive uptake of organic carbon (sucrose), dinitrogen gas, and oxygen into a spherical cell. The uptake of the most limiting resource provided bounds on the growth rate. Growth efficiency was modeled using a balanced stoichiometric network which, following [18], combined half-reactions describing aerobic respiration, nitrogen fixation and synthesis of “biomass” with a prescribed elemental stoichiometry. Mass, electron and energy flow are conserved. The model is, in essence, a highly idealized “Flux Balance Analysis” rooted in the same principles as more detailed, genome-scale metabolic models. The published model [40] had two free parameters, energy conversion efficiency and the permeability of the cell wall, which were constrained by fitting the model to laboratory data when growing diazotrophically in the absence of fixed nitrogen [43]. Nitrogen fixation was assumed only possible when the intracellular oxygen was drawn down to very low concentrations, if necessary, by respiration beyond energetic demands (respiratory protection of nitrogenase, which is highly sensitive to oxygen [9,44]). The model successfully and quantitatively captured the relationship between nitrogen fixation, ambient oxygen concentration and the rate of supply of organic carbon substrate [40].

Here we extend the model to introduce ammonium assimilation as an additional nitrogen source. A complete description of the extended model is provided in the supplementary material. The previous model was simple enough to have a single metabolic configuration. The extended model has three possible metabolic configurations: exclusive diazotrophy, exclusive ammonium assimilation, or a combination of both. In the case of combined ammonium and dinitrogen use, the model organism can use the nitrogen sources in any combined ratio provided the uptake rates will allow it. We define the fraction of total nitrogen assimilation by nitrogen fixation as *f*_*N2*_. The fraction of nitrogen assimilation from ammonium is thus (1 –*f*_*N2*_). Thus an infinite variety of nitrogen sources is possible and we use an optimization approach to determine which combination will result. Since the laboratory data we use to constrain the model are continuous cultures, growth rate cannot be used to optimize “fitness” (as is often assumed). Instead we seek the combination of nitrogen sources which maximizes the standing stock of biomass (or protein) at the given growth (dilution) rate and environmental conditions. Since nitrogen fixation requires additional energy and electrons, relative to ammonium assimilation, it is intuitive that with low organic carbon and high ammonium supply rates, ammonium assimilation will be favored. Here, constrained also by the laboratory data from [34,41] we systematically examine and model the optimal nitrogen assimilation strategy under a variety of carbon and fixed nitrogen supply ratios, as well as a variety of ambient oxygen concentrations.

More specifically, we simulate *Azotobacter vinelandii* grown in a chemostat culture with a supply of ammonium based on laboratory experiments [34,41] for a range of C/N ratios (0–15 mol sucrose mol ammonium^-1^, with constant ammonium at 2.5 mM) and oxygen concentrations (5%, 15%, 30% and 60%), at a dilution rate of 0.15 h^-1^.

Model parameters are adapted from [40], which were either estimated from the literature, or fitted to the data by tuning (two parameters: diffusivity coefficient of the cell membrane layers, *ε*_*m*_, and energy transfer efficiency, *ε*). We employ a higher transfer efficiency, *ε*, for ammonium-uptake based biomass production (*ε* = 0.54) by calibrating the model using laboratory data on microbes grown with glucose and ammonium compiled in [5].

Laboratory data of nitrogen fixation rates, respiration rates and protein concentrations were extracted from [34] (doi: https://doi.org/10.1007/BF00414820) and [41] (doi: https://doi.org/10.1007/BF00414819). The model developed in this paper has been written in Python 3 and uploaded in Zenodo at https://zenodo.org/record/846300 (doi: 10.5281/zenodo.846300).

## Results and discussion

**Fig 3:**
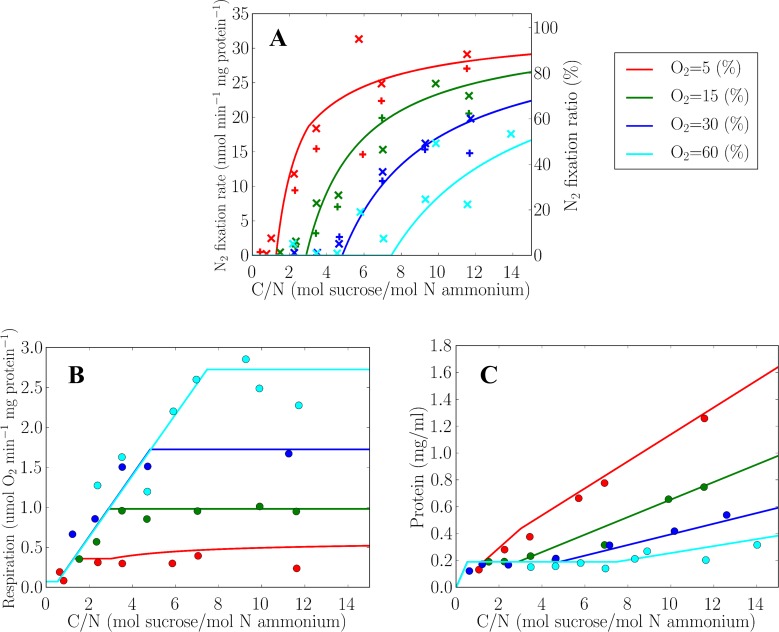
**Simulated protein specific rates of (A) nitrogen fixation (B) respiration and (C) concentrations of protein in continuous cultures of *Azotobacter vinelandii*.** The simulations (solid curves) are compared to laboratory data (points) redrawn from [34,41]. Different colors represent different O_2_ concentration in the culture (see the legend in upper right). Here 100% O_2_ equals 225 μM thus approximately O_2_ saturation under normal air composition at 30°C. At lower to medium C/N in (B) and (C), model results show same values for various O_2_ concentrations. In (A), “+” are data based on acetylene reduction, and “×” are based on the rate of total nitrogen incorporation. The right y-axis in (A) shows the model predicted ratio of nitrogen fixation relative to total nitrogen incorporated into biomass. In both the simulation and the laboratory data, the dilution rate was constant (0.15 h^-1^), and C/N ratio is based on the constant ammonium resource of 2.5 mol m^-3^.

First we simulate the conditions of the laboratory studies and obtain steady-state solutions at a fixed dilution rate of 0.15 h^-1^ across a range of input C/N and ambient oxygen concentrations. Specifically, we impose an ammonium concentration of 2.5 mM in the incoming medium and varied sucrose concentration from 0 to 37.5 mM, giving a C/N supply range of 0–15 (equivalent to an elemental C:N supply ratio of 0–180). Predicted nitrogen fixation and respiration rates are in good agreement with the laboratory data across a range of ambient oxygen concentrations, as shown in Fig 3A and 3B. The model also predicts observed protein concentration in the culture (Fig 3C) as well as biomass concentration (S2 Fig) supporting the model assumption of maximizing culture biomass.

**Fig 4:**
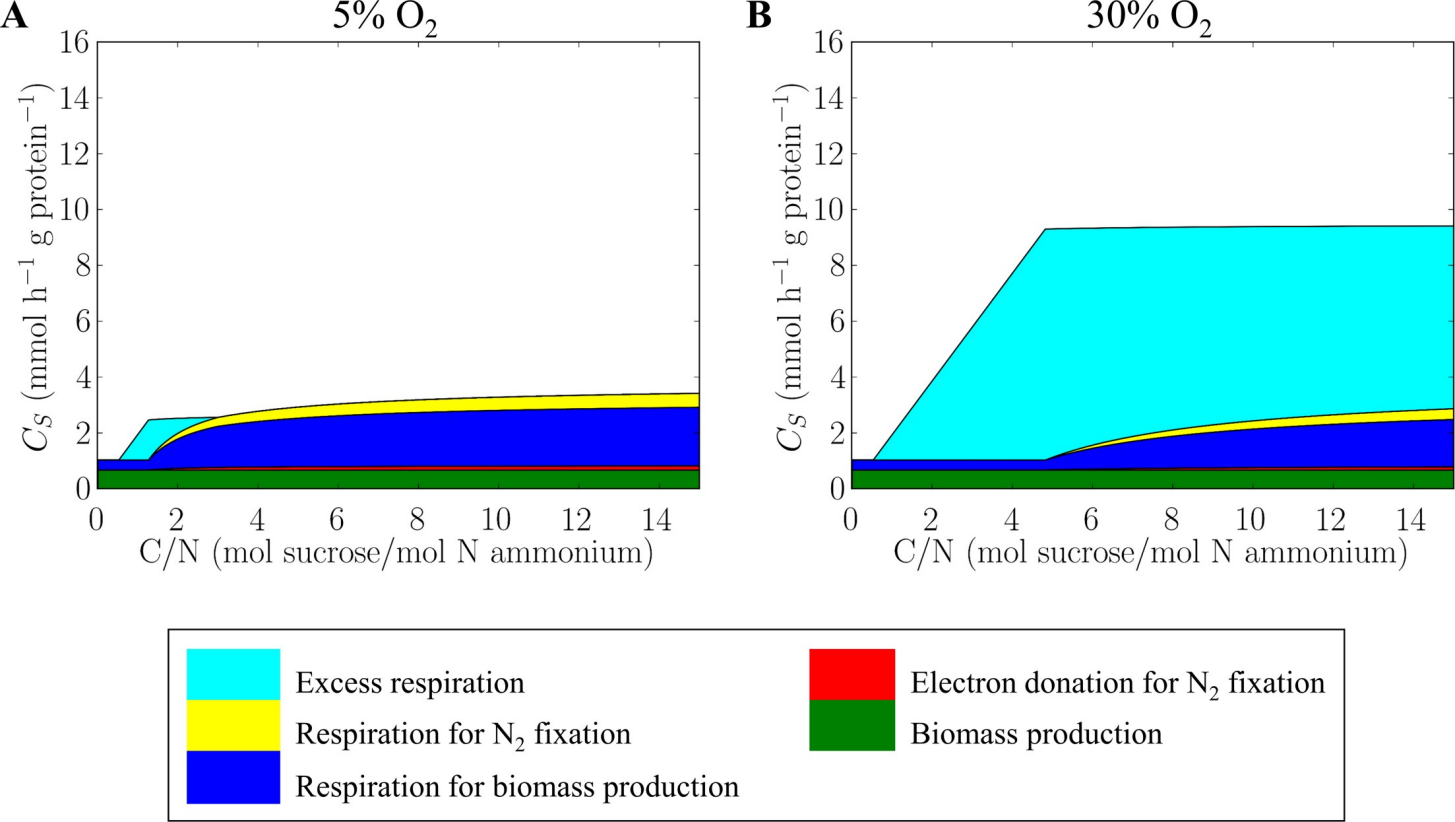
Carbohydrate fluxes for different purposes. (A) 5% O_2_ and (B) 30% O_2_, where 100% O_2_ equals 225 μM thus approximately O_2_ saturation under normal air composition at 30°C. Dilution rate is constant at 0.15 h^-1^. A schematic of carbon allocation is provided in S1 Fig, where the same color scheme is used for each carbohydrate flux.

The model captures salient properties of the observed responses of nitrogen fixation to C/N ratio and oxygen concentration (Fig 3A). Nitrogen fixation occurs only above a threshold C/N ratio ($R_{C/N}^{f}$) and the protein-specific nitrogen fixation rate increases in concert with C/N because carbohydrate in excess of that needed to assimilate the ammonium fuels nitrogen fixation and oxygen scavenging. At a given C/N, the nitrogen fixation rate is negatively impacted by increasing oxygen concentration (Fig 3A) as the cell must devote more carbohydrate towards protective respiration (Fig 3B, Fig 4). At higher C/N ratios, the proportion of the cellular nitrogen requirement accounted for by nitrogen fixation, here defined as *f*_*N2*_, approaches 1 (= 100% in Fig 3A), and the nitrogen fixation rate at different oxygen levels converges to the same maximum rate. Below an oxygen-dependent threshold C/N, nitrogen fixation does not occur either in the laboratory data or model. The model provides an interpretation of these results in terms of three metabolic regimes.

### Nitrogen fixation rates based on three metabolic phases

**Fig 5:**
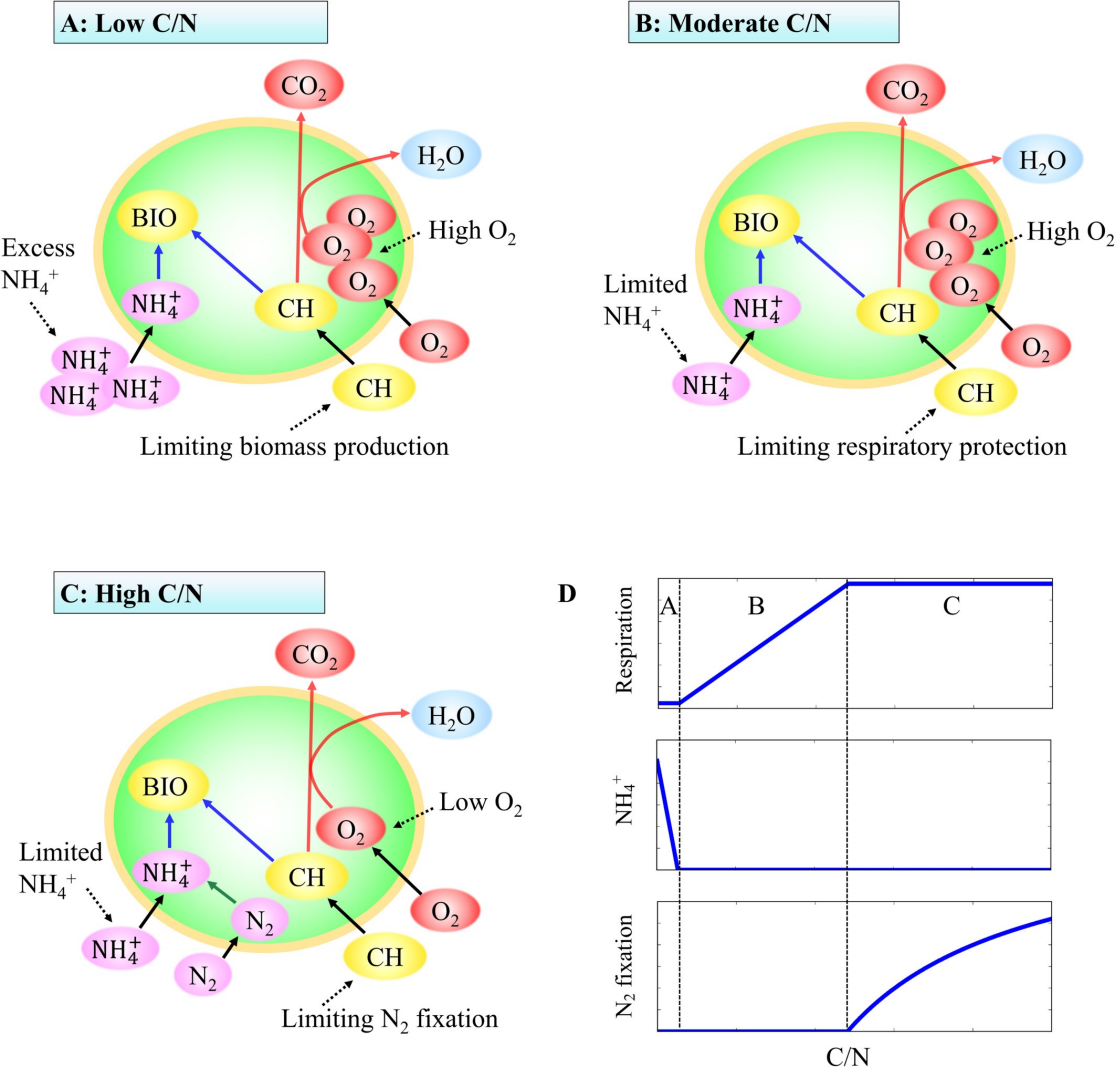
Nitrogen metabolism at different C/N ratios. As C/N ratio increases the cell metabolism shifts from carbohydrate limitation at low C/N supply (A), to ammonium limitation at moderate C/N (B) then to combined ammonium assimilation and nitrogen fixation at high C/N supply which is also carbon limited provided the N_2_ concentration is sufficiently high (C). Black arrows, nutrient uptake; red arrows, respiration; blue arrows, biomass production; green arrow, nitrogen fixation. CH and BIO indicate the intermediate intracellular store of carbohydrate and cellular biomass, respectively. Respiration per unit protein, the ammonium concentration in the medium and nitrogen fixation rate per protein transitions in three phases are shown in (D).

At a given oxygen concentration, based on the maximization of the protein-specific biomass production rate, the model identifies three distinct metabolic regimes corresponding to carbohydrate limited growth (low C/N), ammonium limited growth (moderate C/N), and combined ammonium and dinitrogen-based growth (high C/N), respectively, illustrated schematically in Fig 5.

Below the oxygen-dependent threshold C/N (Fig 5A), carbohydrate is the growth limiting factor and the ammonium in the culture is not fully consumed (Fig 5A and 5D). Since ammonium assimilation requires far less energy and fewer electrons than nitrogen fixation, optimal biomass production occurs in the absence of nitrogen fixation. Hence at low C/N delivery, growth efficiency and specific biomass production rate favor ammonium-based growth. With increased carbohydrate input, the cell density increases leading to a higher protein and biomass concentration (Fig 3C and S2 Fig, lowest C/N).

At moderate C/N ratios (Fig 5B) the cells are ammonium limited. Though all the excess carbohydrate is expended on respiration in excess of energetic demands, it is still insufficient to scavenge oxygen sufficiently to keep the cell cytosol nearly anoxic and protect nitrogenase. Hence nitrogen fixation is still inhibited (see section “High respiration during non-nitrogen fixing phase” for further discussion). Since the incoming ammonium concentration is fixed and ammonium is consumed to an undetectable level [34,41] (and the incoming sucrose concentration varied), the protein and biomass concentration stays constant in both laboratory experiments and simulations (Fig 3C and S2 Fig, constant lines).

At high C/N ratio (Fig 5C), due to excess carbohydrate relative to ammonium, ammonium in the medium is drawn down to its subsistence concentration (Fig 5D) and there is sufficient additional carbon with which to completely scavenge oxygen (Flat part in Figs 3B and 4) and fuel nitrogen fixation (Figs 3A, 4 and 5D). In this regime, growth is based on both ammonium assimilation and nitrogen fixation and the modeled nitrogen fixation rate increases non-linearly with the C/N ratio (Fig 3A), consistent with the laboratory data [34]. The experimental data are consistent with a model in which the cells allocate carbohydrate to ammonium assimilation and nitrogen fixation (with its attendant respiratory protection) in a manner that maximizes the standing stock of biomass and biomass production. Increasing carbohydrate resource leads to higher nitrogen fixation increasing protein and biomass concentration in the culture (higher end of C/N in Fig 3C and S2 Fig).

The diffusion of oxygen into the cells negatively influences the nitrogen fixation rate. As the ambient oxygen concentration increases, so does respiration rate (Fig 3B) as carbon is channeled into respiratory protection (Fig 4), at the expense of nitrogen fixation (Fig 3A). At an oxygen concentration at 60% of saturation (here 100% saturation equals 225 μM; about 21% atmospheric composition at a temperature of 30°C), more than half of nitrogen for growth is supplied by ammonium if the C/N (sucrose/ammonium) supply ratio is lower than 14 (Fig 3A). On the other hand, at a low oxygen saturation of 5%, nitrogen fixation accounts for about 80% of nitrogen in biomass at C/N ratios higher than 8 (Fig 3A). The threshold C/N above which nitrogen fixation occurs is oxygen dependent (Fig 3A, Fig 4). The higher the ambient oxygen concentration the greater the required expenditure on respiratory protection, and the greater the C/N threshold for nitrogen fixation. This is qualitatively and quantitatively captured by the idealized metabolic model.

### Nitrogen fixation as a function of carbohydrate, oxygen, and ammonium

**Fig 6:**
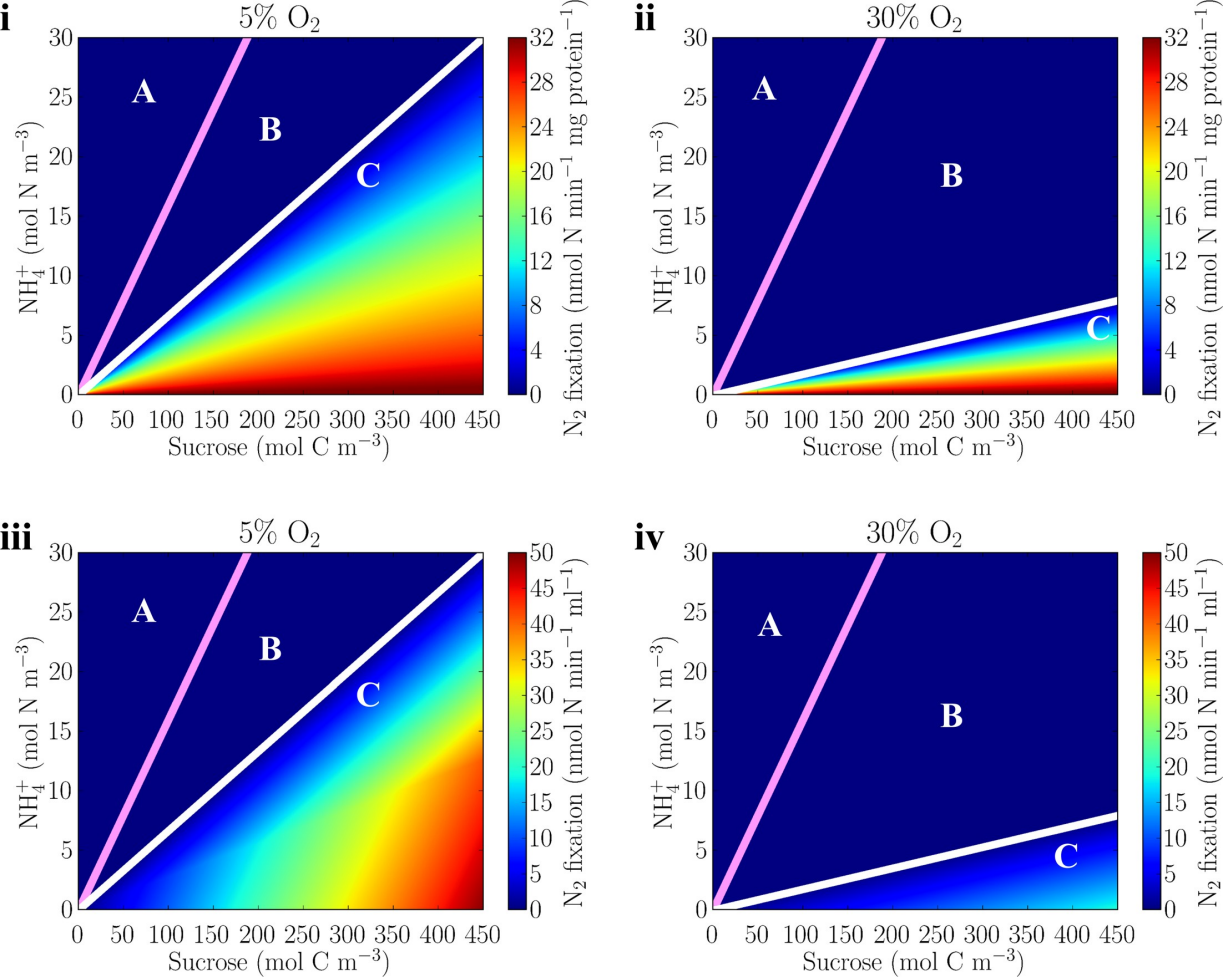
Nitrogen fixation rate under different sucrose, ammonium, and oxygen regimes. Nitrogen fixation shown per protein (i and ii) and per volume (iii and iv) at oxygen saturations of 5% (i and iii) and 30% (ii and iv). Dilution rate is constant at 0.15 h^-1^. Pink and white lines indicate the transitions from carbohydrate limited (A) to ammonium limited (B), and from ammonium assimilation only (A, B) to ammonium assimilation and nitrogen fixation (C) regimes respectively; Regimes (A) ~ (C) here represent cellular states (A) ~ (C) defined in Fig 3 respectively. Here 100% O_2_ equals 225 μM thus approximately O_2_ saturation under normal air composition at 30°C. The net growth isocline (ZNGI) is much smaller than the scale of the axes here [34,41] and it is assumed small in this model; both ammonium and sucrose concentrations in the culture are much smaller than those in the incoming medium in the laboratory studies [34,41].

The laboratory data and simulations discussed above considered a constant ammonium input (ammonium concentration of 2.5 mM and dilution rate of 0.15 hr^-1^). Having demonstrated the consistency of the model and its parameters with these data, we now extrapolate beyond the range of laboratory studies to consider the variation of nitrogen fixation in *Azotobacter vinelandii* over a range of both ammonium and sucrose input concentrations, and at two levels of oxygen saturation: 5% and 30% (Fig 6).

As above, the nitrogen fixation rate per unit protein is controlled by the C/N ratio and by oxygen concentration (Fig 6i and 6ii). Note that contours of constant C/N have constant per protein nitrogen fixation rates. The critical C/N ratio for the onset of nitrogen fixation (white line, transition from regime B to regime C) increases with the oxygen concentration but even in a high oxygen environment (Fig 6ii), as long as the C/N ratio is sufficiently high, the model predicts high nitrogen fixation rate per protein.

Nitrogen fixation rate per unit volume of culture, however, varies along lines of constant C/N (compare regime C in Fig 6i and 6iii) since higher carbohydrate supply supports higher densities of cells at the same dilution rate (S3 Fig). Since ammonium does not suppress cell density but rather increases it in some regimes (S3 Fig), the negative influence of ammonium on nitrogen fixation per volume is relatively limited. The nitrogen fixation rate per volume is more severely suppressed by oxygen (Fig 6iii vs. 6iv); at 30% oxygen, the nitrogen fixation rate per volume is significantly decreased due to expenditure on respiratory protection which lowers the cell concentration (S3 Fig).

### High respiration during non-nitrogen fixing phase

**Fig 7:**
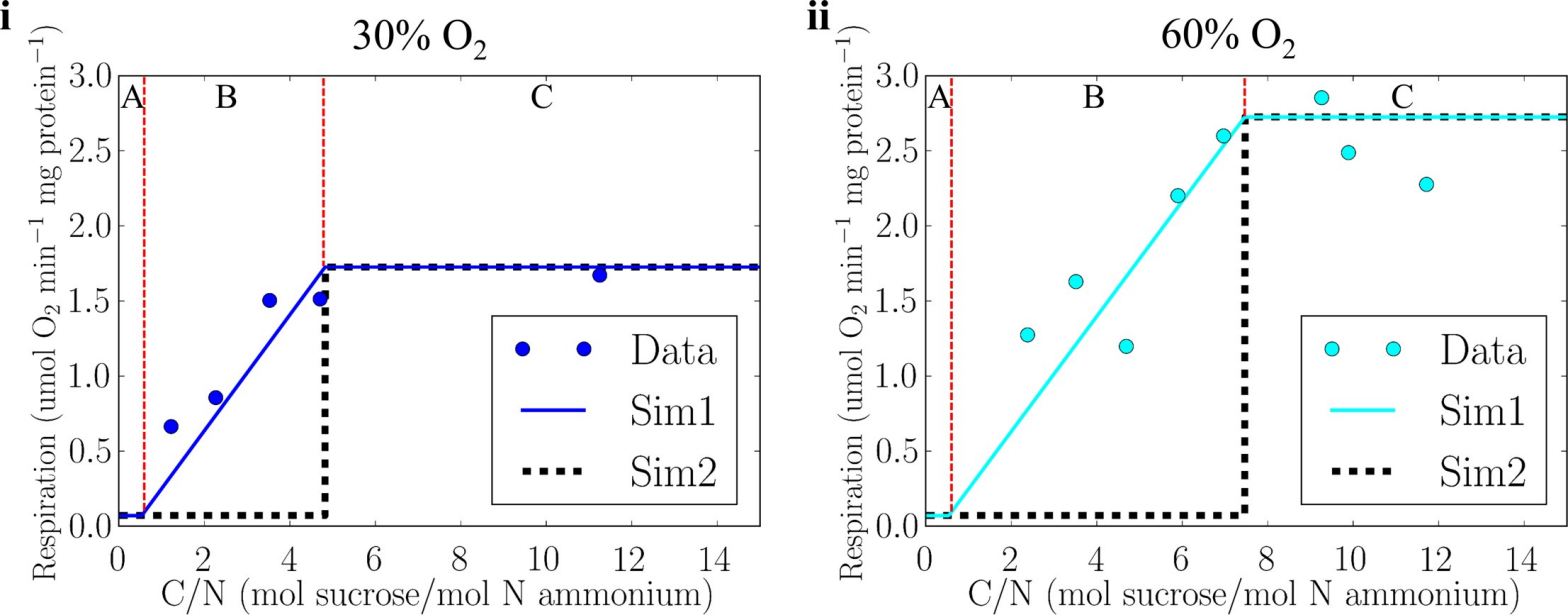
Respiration per protein as a function of C/N. Data (points) are shown as well as results of two simulation models. In one simulation (Sim1) energetically excess respiration can occur both during nitrogen fixation and during nitrogen limited growth on ammonium. In the second model (Sim2) excess respiration is allowed only during nitrogen fixation. Sim1 and Sim2 produce same amount of biomass. (i) 30% O_2_ and (ii) 60% O_2_. Solids lines and points are same as Fig 3B. The dilution rate is constant of 0.15 h^-1^, and red dashed lines show borders between different regimes (A: Carbohydrate limited, B: Ammonium limited, C: Nitrogen fixing as defined in Fig 5). Here 100% O_2_ equals 225 μM thus approximately O_2_ saturation under normal air composition at 30°C.

Nitrogen fixation requires low intra-cellular oxygen concentrations since nitrogenase is sensitive to oxygen. *Azotobacter* manages intracellular oxygen using respiratory protection [7,11] which has also been indicated in other nitrogen fixers including *Crocosphaera*, *Trichodesmium* and *Anabaena* [13,15,45,46]. Interestingly, comparison of models and data (Fig 7) suggests *Azotobacter* constitutively respires any carbohydrate in excess of the energetic demand for synthesizing biomass, even if it is insufficient to fully deplete intracellular oxygen.

To examine the level of respiration during ammonium limited growth, we compare two simulations. One simulation (Sim1) assumes that all ‘excess’ carbohydrate will be respired under all growth conditions: that is, the metabolic configuration is identified that maximizes biomass concentration. In the other simulation (Sim2), ‘excess’ respiration only occurs when the cells are fixing nitrogen, reflecting the possibility that respiratory protection is regulated and repressed when the cell is not actively fixing nitrogen. We find that model Sim1 predicts an increase in respiration with increasing C/N, consistent with observations (Compare Sim1 and data in Fig 7i and 7ii), whereas Sim2 substantially underestimates rates of respiration during growth on ammonium (Compare Sim2 and data in Fig 7i and 7ii). The concordance of Sim1 with laboratory measurements suggests that oxygen-scavenging respiration is performed even when the cells are not able to fix nitrogen.

There are several possible explanations for these observations. One is that nitrogen fixation is so central to the biology and ecology of these organisms, that they perform excess respiration (respiratory protection) constitutively, at least to the point where it does not have a growth cost due to limitation by carbon. This might reflect adaptation of other enzymes or intracellular processes to low oxygen conditions, or might reduce the need for regulation of respiratory protection pathways. The latter might be advantageous particularly in dynamic or patchy environments, and might allow cells to preserve nitrogenase when they are not fixing nitrogen. In this case, cells might be able to commence nitrogen fixation rapidly when conditions allow [12].

### Wider implications

#### Simultaneous N_2_ and fixed-N assimilation and relative fitness

The experimental results of [34,41] demonstrate that nitrogen fixing organisms can, and will, simultaneously use both fixed and molecular nitrogen as nitrogen source. In their experiments, *Azotobacter vinelandii* drew down ammonium concentrations to undetectable levels even when fixing nitrogen, provided the supply of organic carbon was sufficient relative to the supply of fixed nitrogen. Our model captures this threshold and suggests that this is consistent with a metabolic organization which maximizes the per protein biomass production rate [47]. This is also consistent with numerous lines of evidence showing that, while the availability of fixed nitrogen can suppress nitrogen fixation in diazotrophs, it does not always do so [21]. Many of these examples are phototrophic nitrogen fixers in marine environments [21]. In the oligotrophic surface ocean, light and energy are in excess supply relative to nitrogen (analogous to the excess organic carbon supply to *Azotobacter*). Under these circumstances, where the direct and indirect (oxygen management) costs of nitrogen fixation by phototrophs can be easily met, it is still a fitness advantage to utilize both N_2_ and fixed nitrogen: by doing so, the diazotrophs increase its overall growth efficiency as assimilating fixed nitrogen is energetically cheaper. It also denies some of the limiting nitrogen resource for the non-diazotrophs, effectively increasing the size of the niche, or potential range, for the nitrogen fixer at the expense of the non-diazotrophs. A simple theoretical perspective to demonstrate this is outlined in the Supplementary Material (S2 Text).

#### Inferences on the viability of marine heterotrophic nitrogen fixation

We chose to model a heterotrophic, diazotrophic soil bacterium, *Azotobacter vinelandii*, because of the comprehensive laboratory data sets available. Analogous organisms are present in the oceans. Nitrogenase genes associated with heterotrophic bacteria are almost ubiquitous in marine waters and commonly dominate the *nifH* gene pool throughout the water column when evaluated from PCR amplicon libraries [48,49]. While the rates of nitrogen fixation associated with these organisms is generally considered to be lower than that of photoautotrophic nitrogen fixers, there are observations of heterotrophs actively fixing nitrogen both in surface and deeper waters [50–54]. Whether or not their contribution to global nitrogen fixation is large, the existence of these organisms suggests an ecological niche and an evolutionary advantage. However, it is still not clear how exactly these organisms carry out nitrogen fixation in oxygenated marine environments. Recent studies show that a model organism BAL361 responds to oxygen and ammonium similarly to *Azotobacter vinelandii* [35,55] and here, we apply our model to consider the potential limitations on their niche.

Consider the critical atomic ratio of carbon to nitrogen in the substrate: $R_{C:N}^{f}$ (= 12 $R_{C/N}^{f}$). (mol C mol N^-1^) which for sucrose-fueled *Azotobacter* lies between ~15 at an oxygen concentration of 5% of saturation, and ~100 at 60% of saturation. This C:N is higher than that of most observed particulate organic matter in marine systems [56,57], suggesting that heterotrophic nitrogen fixation will be unlikely to occur.

However, using the model we predict $R_{C:N}^{f}$ to be as low as 6.5 at 1% oxygen saturation; lower than many observed samples of particulate organic matter [56,57] as well as the observed elemental ratio of dissolved organic matter, DOM [56,58,59]. The model also suggests that at this oxygen concentration respiratory protection is not necessary since energetically balanced respiration for nitrogen acquisition and biosynthesis fully deplete the intracellular oxygen. This implies that heterotrophic nitrogen fixation may occur even in the presence of ammonium at the low oxygen concentrations which can exist within particles due to active heterotrophic respiration [60]. The composition and nutritional value of natural DOM and POM is poorly characterized and variable, and the analogy between *Azotobacter* and marine heterotrophic diazotrophs like BAL361 is not proven. However, the relationships described by the model above provide clear hypotheses which could be tested with laboratory cultures of marine organisms, employing organic matter of known stoichiometry and controlled oxygen conditions.

### Summary

Using a simplified metabolic model, we simulated continuous cultures of the heterotrophic nitrogen fixer *Azotobacter vinelandii*, identifying and quantifying relationships between the availability of fixed nitrogen and nitrogen-fixation. We find that using both nitrogen sources is optimal for the diazotroph when energy and other resources allow and identify the transitions between nitrogen and carbon limited regimes. We show that *Azotobacter* uses organic carbon sources in excess of the requirements for respiratory protection even when there is insufficient excess carbon to deplete intra-cellular oxygen and enable nitrogen fixation. The model suggests that using both fixed nitrogen and dinitrogen gas, marine diazotrophs increases their fitness and the size of their fundamental niche. Finally, the model provides a testable hypothesis that heterotrophic nitrogen fixation may occur in marine particles despite the presence of ammonium.

## Supporting information

S1 Text Model details. (PDF)

S2 Text Fitness advantage of using fixed nitrogen and dinitrogen. (PDF)

S1 Fig Schematic of detailed carbon fluxes in a modeled cell. (PDF)

S2 Fig Simulated concentrations of biomass in continuous cultures of *Azotobacter vinelandii*. (PDF)

S3 Fig Cell density proxied by protein concentration for various sucrose and ammonium resources with different oxygen saturations. (PDF)

## References

[1] Gruber N, Galloway JN. An Earth-system perspective of the global nitrogen cycle. Nature. 2008;451: 293–296. 10.1038/nature06592 18202647 10.1038/nature06592

[2] Falkowski PG. Evolution of the nitrogen cycle and its influence on the biological sequestration of CO_2_ in the ocean. Nature. 1997;387: 272–275. 10.1038/387272a0

[3] Falkowski P, Scholes RJ, Boyle E, Canadell J, Canfield D, Elser J, et al The global carbon cycle: A test of our knowledge of Earth as a system. Science. 2000;290: 291–297. 11030643 10.1126/science.290.5490.291

[4] Karl D, Michaels A, Bergman B, Capone D, Carpenter E, Letelier R, et al Dinitrogen fixation in the world’s oceans. Biogeochemistry. 2002;57/58: 47–98.

[5] Heijnen JJ, Roels JA. A macroscopic model describing yield and maintenance relationships in aerobic fermentation processes. Biotechnol Bioeng. 1981;23: 739–763.

[6] Oelze J. Respiratory protection of nitrogenase in *Azotobacter* species: is a widely held hypothesis unequivocally supported by experimental evidence? FEMS Microbiol Rev. 2000;24: 321–333. 10978541 10.1111/j.1574-6976.2000.tb00545.x

[7] Poole RK, Hill S. Respiratory protection of nitrogenase activity in *Azotobacter vinelandii*-Roles of the terminal oxidases. Biosci Rep. 1997;17: 303–317. 9337485 10.1023/a:1027336712748

[8] Robson RL, Postgate JR. Oxygen and hydrogen in biological nitrogen fixation. Annu Rev Microbiol. 1980;34: 183–207. 10.1146/annurev.mi.34.100180.001151 6776883 10.1146/annurev.mi.34.100180.001151

[9] Gallon JR. The oxygen sensitivity of nitrogenase: a problem for biochemists and micro-organisms. Trends Biochem Sci. 1981;6: 19–23. 10.1016/0968-0004(81)90008-6

[10] Wang D, Xu A, Elmerich C, Ma LZ. Biofilm formation enables free-living nitrogen-fixing rhizobacteria to fix nitrogen under aerobic conditions. ISME J. Nature Publishing Group; 2017;24: 10.1038/ismej.2017.30 10.1038/ismej.2017.30 PMC5520150 28338674

[11] Dalton H, Postgate JR. Effect of oxygen on growth of *Azotobacter chroococcum* in batch and continuous cultures. J Gen Microbiol. 1969;54: 463–473. 10.1099/00221287-54-3-463 5709283

[12] Kuhla J, Oelze J. Dependence of nitrogenase switch-off upon oxygen stress on the nitrogenase activity in *Azotobacter vinelandii*. J Bacteriol. 1988;170: 5325–5329. 3182730 10.1128/jb.170.11.5325-5329.1988 PMC211608

[13] Großkopf T, LaRoche J. Direct and indirect costs of dinitrogen fixation in Crocosphaera watsonii WH8501 and possible implications for the nitrogen cycle. Front Microbiol. 2012;3: 10.3389/fmicb.2012.00236 22833737 10.3389/fmicb.2012.00236 PMC3401090

[14] Berman-Frank I, Lundgren P, Falkowski P. Nitrogen fixation and photosynthetic oxygen evolution in cyanobacteria. Res Microbiol. 2003;154: 157–164. 10.1016/S0923-2508(03)00029-9 12706503 10.1016/S0923-2508(03)00029-9

[15] Berman-Frank I, Lundgren P, Chen Y-B, Küpper H, Kolber Z, Bergman B, et al Segregation of Nitrogen Fixation and Oxygenic Photosynthesis in the Marine Cyanobacterium Trichodesmium. Science. 2001;294: 1534–1537. 10.1126/science.1064082 11711677 10.1126/science.1064082

[16] Fay P. Oxygen relations of nitrogen fixation in cyanobacteria. Microbiol Rev. 1992;56: 340–373. 1620069 10.1128/mr.56.2.340-373.1992 PMC372871

[17] Staal M, Meysman FJR, Stal LJ. Temperature excludes N_2_-fixing heterocystous cyanobacteria in the tropical oceans. Nature. 2003;425: 504–507. 10.1038/nature01999 14523445 10.1038/nature01999

[18] Rittmann BE, McCarty PL. Stoichiometry and bacterial energetics In: Environmental Biotechnology: Principles and Applications. McGraw-Hill: New York McGraw-Hill; 2001 pp. 126–164.

[19] Laane C, Krone W, Konings W, Haaker H, Veeger C. Short-term effect of ammonium chloride on nitrogen fixation by *Azotobacter vinelandii* and by bacteroids of *Rhizobium leguminosarum*. Eur J Biochem. 1980;103: 39–46. 10.1111/j.1432-1033.1980.tb04286.x 6928406 10.1111/j.1432-1033.1980.tb04286.x

[20] Dixon R, Kahn D. Genetic regulation of biological nitrogen fixation. Nat Rev Microbiol. 2004;2: 621–631. 10.1038/nrmicro954 15263897 10.1038/nrmicro954

[21] Knapp AN. The sensitivity of marine N_2_ fixation to dissolved inorganic nitrogen. Front Microbiol. 2012;3: 10.3389/fmicb.2012.00374 23091472 10.3389/fmicb.2012.00374 PMC3476826

[22] Fernandez C, Far L1, Ulloa O. Nitrogen fixation in denitrified marine waters. PLOS ONE. 2011;6 10.1371/journal.pone.0020539 21687726 10.1371/journal.pone.0020539 PMC3110191

[23] Voss M, Croot P, Lochte K, Mills M, Peeken I. Patterns of nitrogen fixation along 10° N in the tropical Atlantic. Geophys Res Lett. 2004;31: 10.1029/2004GL020127

[24] Welsh DT, de Wit R, Herbert RA. Seasonal variations in nitrogen-fixation (acetylene reduction) and sulphate-reduction rates in the rhizosphere of *Zoster noltii*: nitrogen fixation by sulphate-reducing bacteria. Mar Biol. 1996;125: 619–628. 10.1007/BF00349243

[25] Currin CA, Joye SB, Paerl HW. Diel rates of N_2_-fixation and denitrification in a transplanted *Spartina alterniflora* marsh: Implications for N-flux dynamics. Estuar Coast Shelf Sci. 1996;42: 597–616.

[26] Haines JR, Atlas RM, Griffiths RP, Morita RY. Denitrification and nitrogen fixation in Alaskan continental shelf sediments. Appl Environ Microbiol. 1981;41: 412–421. 16345716 10.1128/aem.41.2.412-421.1981 PMC243709

[27] Bertics VJ, Sohm JA, Treude T, Chow C-ET, Capone DG, Fuhrman JA, et al Burrowing deeper into benthic nitrogen cycling: the impact of bioturbation on nitrogen fixation coupled to sulfate reduction. Mar Ecol Prog Ser. 2010;409: 10.3354/meps08639

[28] Capone DG. Nitrogen fixation (acetylene reduction) by rhizosphere sediments of the eelgrass Zostera marina. Mar Ecol. 1982;10: 67–75.

[29] Casselman ME, Patrick WH, DeLaune RD. Nitrogen fixation in a gulf coast salt marsh. Soil Sci Soc Am J. 1981;45: 51–56.

[30] Hanson RB. Nitrogen fixation (acetylene reduction) in a salt marsh amended with sewage sludge and organic carbon and nitrogen compounds. Appl Environ Microbiol. 1977;33: 846–852. 16345239 10.1128/aem.33.4.846-852.1977 PMC170778

[31] Capone DG. Benthic nitrogen fixation In: Nitrogen Cycling in Coastal Marine Environments. John Willey and Sons, New York; 1988 pp. 85–123.

[32] Holl CM, Montoya J. Interactions between nitrate uptake and nitrogen fixation in continuous cultures of the marine diazotroph *Trichodesmium* (cyanobacteria). J Phycol. 2005;41: 1178–1183. 10.1111/j.1529-8817.2005.00146.x

[33] Fu F-X, Bell PRF. Factors affecting N_2_ fixation by the cyanobacterium *Trichodesmium* sp. GBRTRLI101. FEMS Microbiol Ecol. 2003;45: 203–209. 10.1016/S0168-6496(03)00157-0 19719631 10.1016/S0168-6496(03)00157-0

[34] Bühler T, Sann R, Monter U, Dingier C, Kuhla J, Oelze J. Control of dinitrogen fixation in ammonium-assimilating cultures of *Azotobacter vinelandii*. Arch Microbiol. 1987;148: 247–251. 10.1007/BF00414820

[35] Bentzon-Tilia M, Severin I, Hansen LH, Riemann L. Genomics and ecophysiology of heterotrophic nitrogen-fixing bacteria isolated from estuarine surface water. mBio. 2015;6: e00929-15-15. 10.1128/mBio.00929-15.Editor 10.1128/mBio.00929-15 PMC4495170 26152586

[36] Moore JK, Doney SC, Lindsay K. Upper ocean ecosystem dynamics and iron cycling in a global three-dimensional model. Global Biogeochem Cycles. 2004;18: GB4028, 10.1029/2004GB002220

[37] Le Quéré C, Harrison SP, Colin Prentice I, Buitenhuis ET, Aumont O, Bopp L, et al Ecosystem dynamics based on plankton functional types for global ocean biogeochemistry models. Glob Chang Biol. 2005;11: 2016–2040. 10.1111/j.1365-2486.2005.01004.x

[38] Stukel MR, Coles VJ, Brooks MT, Hood RR. Top-down, bottom-up and physical controls on diatom-diazotroph assemblage growth in the Amazon River plume. Biogeosciences. 2014;11: 3259–3278. 10.5194/bg-11-3259-2014

[39] Dutkiewicz S, Ward BA, Scott JR, Follows MJ. Understanding predicted shifts in diazotroph biogeography using resource competition theory. Biogeosciences Discuss. 2014;11: 7113–7149. 10.5194/bgd-11-7113-2014

[40] Inomura K, Bragg J, Follows MJ. A quantitative analysis of the direct and indirect costs of nitrogen fixation: a model based on Azotobacter vinelandii. ISME J. 2017;11: 165–175. 10.1038/ismej.2016.97 27740611 10.1038/ismej.2016.97 PMC5315487

[41] Bühler T, Monter U, Sann R, Kuhla J, Dingier C, Oelze J. Control of respiration and growth yield in ammonium-assimilating cultures of *Azotobacter vinelandii*. Arch Microbiol. 1987;148: 242–246. 10.1007/BF00414819

[42] Noar JD, Bruno-Bárcena JM. *Azotobacter vinelandii*: the source of 100 years of discoveries and many more to come. Microbiology. 2018;164: 421–436. 10.1099/mic.0.000643 29533747 10.1099/mic.0.000643

[43] Kuhla J, Oelze J. Dependency of growth yield, maintenance and *K*_s_-values on the dissolved oxygen concentration in continuous cultures of *Azotobacter vinelandii*. Arch Microbiol. 1988;149: 509–514.

[44] Wang ZC, Burns A, Watt GD. Complex formation and O_2_ sensitivity of *Azotobacter vinelandii* nitrogenase and its component proteins. Biochemistry. 1985;24: 214–221. 2986674 10.1021/bi00322a031

[45] Peschek GA, Villgrater K, Wastyn M. “Respiratory protection” of the nitrogenase in dinitrogen-fixing cyanobacteria. Plant Soil. 1991;48: 411–418.

[46] Murry MA, Wolk CP. Evidence that the barrier to the penetration of oxygen into heterocysts depends upon two layers of the cell envelope. Arch Microbiol. 1989;151: 469–474.

[47] Orth JD, Thiele I, Palsson BO. What is flux balance analysis? Nat Biotech. 2010;28: 245–248. 10.1038/nbt.1614 20212490 10.1038/nbt.1614 PMC3108565

[48] Riemann L, Farnelid H, Steward GF. Nitrogenase genes in non-cyanobacterial plankton: Prevalence, diversity and regulation in marine waters. Aquat Microb Ecol. 2010;61: 235–247. 10.3354/ame01431

[49] Farnelid H, Andersson AF, Bertilsson S, Al-soud WA, Hansen LH. Nitrogenase gene amplicons from global marine surface waters are dominated by genes of non-cyanobacteria. PLOS ONE. 2011;6: e19223 10.1371/journal.pone.0019223 21559425 10.1371/journal.pone.0019223 PMC3084785

[50] Rahav E, Bar-Zeev E, Ohayon S, Elifantz H, Belkin N, Herut B, et al Dinitrogen fixation in aphotic oxygenated marine environments. Front Microbiol. 2013;4: 10.3389/fmicb.2013.00227 23986748 10.3389/fmicb.2013.00227 PMC3753716

[51] Turk-kubo KA, Karamchandani M, Capone DG, Zehr JP. The paradox of marine heterotrophic nitrogen fixation: abundances of heterotrophic diazotrophs do not account for nitrogen fixation rates in the Eastern Tropical South Pacific. Environ Microbiol. 2014;16: 3095–3114. 10.1111/1462-2920.12346 24286454 10.1111/1462-2920.12346

[52] Benavides M, Moisander PH, Berthelot H, Dittmar T, Grosso O, Bonnet S. Mesopelagic N_2_ fixation related to organic matter composition in the Solomon and Bismarck Seas (Southwest Pacific). PLOS ONE. 2015;10: e0143775 10.1371/journal.pone.0143775 26659074 10.1371/journal.pone.0143775 PMC4684240

[53] Bonnet S, Dekaezemacker J, Turk-kubo KA, Moutin T, Hamersley RM, Grosso O, et al Aphotic N*2* fixation in the Eastern Tropical South Pacific Ocean. PLOS ONE. 2013;8: e81265 10.1371/journal.pone.0081265 24349048 10.1371/journal.pone.0081265 PMC3861260

[54] Benavides M, Bonnet S, Berman-frank I, Riemann L. Deep into oceanic N2 fixation. Front Mar Sci. 2018;5: 10.3389/fmars.2018.00043 The.

[55] Paerl RW, Hansen T, Henriksen NNSE, Olesen AK, Riemann L. N-fixation and related O_2_ constraints on model marine diazotroph Pseudomonas stutzeri BAL361. Aquat Microb Ecol. 2018;81: 125–136. 10.3354/ame01867

[56] Williams PM, Carlucci AF, Oison R. A deep profile of some biologically important properties in the central North Pacifie gyre. Oceanol Acta. 1980;3: 471–476.

[57] Laws EA, DiTullio GR, Betzer PR, Karl DM, Carder KL. Autotrophic production and elemental fluxes at 26°N, 155°W in the North Pacific subtropical gyre. Deep Res. 1989;36: 103–120.

[58] Benner R, Pakulski D, McCarthy M, Hedges JI, Hatcher PG. Bulk chemical characteristics of dissolved organic matter in the ocean. Science. 1992;255: 1561–1564. 10.1126/science.255.5051.1561 17820170 10.1126/science.255.5051.1561

[59] Hopkinson CS, Vallino JJ. Efficient export of carbon to the deep ocean through dissolved organic matter. Nature. 2005;433: 142–145. 10.1038/nature03191 15650735 10.1038/nature03191

[60] Klawonn I, Bonaglia S, Brüchert V, Ploug H. Aerobic and anaerobic nitrogen transformation processes in N_2_-fixing cyanobacterial aggregates. ISME J. 2015;9: 1456–1466. 10.1038/ismej.2014.232 25575306 10.1038/ismej.2014.232 PMC4438332

