# Supplementary material for: A quantitative model of nitrogen fixation in the presence of ammonium
Source: PLoS One. 2018 Nov 29;13(11):e0208282. doi: 10.1371/journal.pone.0208282 (PMC6264846; doi:10.1371/journal.pone.0208282)
Supplement: S1 Text — (PDF) [file pone.0208282.s001.pdf]

Model Details

We discuss three metabolic regimes that emerge in the model solutions based on the C/N ratio of the medium: carbohydrate limited, ammonium limited, and nitrogen fixation. We determine the appropriate or optimal metabolic regime by computing the cell density and metabolic fluxes such as respiration, nitrogen fixation and biomass synthesis in each of the possible metabolic configurations. Here we briefly describe explain how the cell densities are evaluated and how the optimal metabolic configuration is determined. Then we describe the method for solving the full state of the population. Fundamental reactions used for stoichiometric parameterization is listed in S1 Table. Nomenclature is provided in S2 Table.

Obtaining cell densities

Cell density is a function of the metabolic state of the population which is, in turn, a function of the resource limiting population growth. We evaluate the cell density for each potential limiting factor:

Carbohydrate limited case. To obtain the cell density, we consider the conservation of carbohydrate in the chemostat culture as described in [1]:

$$\frac{d[CH]}{dt} = D\left([CH]_{IN} - [CH]\right) - V_{CH} X_{CH} \quad \text{(S1)}$$

where $[CH]$ (mol C m^{-3}) is carbohydrate concentration in the culture, t (s) is time, $[CH]_{IN}$ (mol C m^{-3}) is the carbohydrate concentration in the incoming medium, V_{CH} (mol C cell^{-1} s^{-1}) is the averaged carbohydrate uptake rate per cell (mol C cell^{-1} s^{-1}), and X_{CH} is the cell density based on carbohydrate limitation. The balance in the intracellular carbohydrate CH_C (mol C cell^{-1}) is as follows:

$$\frac{dCH_C}{dt} = V_{CH} - \lambda_S \left(1 + E_{CH}\right) \quad \text{(S2)}$$

where λ_S is biomass production (mol C cell^{-1} s^{-1}) and E_{CH} is the ratio of biomass production to CO_2 production under carbohydrate limitation (see the section "Obtaining stoichiometric parameters" for E_{CH}). Assuming a fixed mean cellular carbon quota, Q_C, λ_S is represented by the product of Q_C and growth rate μ (s^{-1}):

$$\lambda_S = Q_C \mu \tag{S3}$$

In the chemostat, steady state is maintained (time variation terms = 0) and growth rate is balanced by dilution rate ($D = \mu$). Thus, from (S1),

$$X_{CH} = \frac{D\left([CH]_{IN} - [CH]\right)}{V_{CH}} \tag{S4}$$

from (S2),

$$V_{CH} = \lambda_S \left(1 + E_{CH}\right) \tag{S5}$$

and from (S3),

$$\lambda_S = Q_C D \tag{S6}$$

By arranging (S4)~(S6), we arrive at an expression for X_{CH}:

$$X_{CH} = \frac{[CH]_{IN} - [CH]}{Q_C \left(1 + E_{CH}\right)} \tag{S7}$$

Under carbohydrate limited condition, the carbohydrate in the medium is consumed and drawn down to the subsistence concentration [2,3], such that $[CH]_{IN} >> [CH]$, which further simplifies (S7):

$$X_{CH} = \frac{[CH]_{IN}}{Q_C \left(1 + E_{CH}\right)} \tag{S8}$$

Ammonium limited case. When the growth of the cell is limited by ammonium we compute the cell density by considering the governing equation for the ammonium concentration in the chemostat:

$$\frac{d[NH_4^+]}{dt} = D\left([NH_4^+]_{IN} - [NH_4^+]\right) - X_{NH4+}V_{NH4+} \tag{S9}$$

where $[NH_4^+]_{IN}$ (mol N m^{-3}) is the ammonium concentration in the incoming medium, D (s^{-1}) is the dilution rate, $[NH_4^+]$ (mol N m^{-3}) is the ammonium concentration in the culture, X_{NH4+} (cell m^{-3}) is the cell density based on ammonium limitation, and V_{NH4+} (mol N cell^{-1} s^{-1}) is the averaged ammonium uptake rate. In the steady state (thus time variation term = 0), (S9) can be solved for X_{NH4+}:

$$X_{NH4+} = \frac{D\left([NH_4^+]_{IN} - [NH_4^+]\right)}{V_{NH4+}} \tag{S10}$$

and under ammonium limitation, ammonium is consumed to the subsistence level as in [2]; thus $[NH_4^+]_{IN} >> [NH_4^+]$ leading to

$$X_{NH4+} = \frac{D[NH_4^+]_{IN}}{V_{NH4+}} \tag{S11}$$

To estimate $V_{NH_4^+}$ we consider the balance of the ammonium pool in the cell:

$$\frac{dNH_{4\,Cell}^+}{dt} = V_{NH4+} - \lambda_S Y_{Bio}^{N:C} \tag{S12}$$

where $NH_{4\,Cell}^+$ (mol N cell^{-1}) is the cellular ammonium quota, and $Y_{Bio}^{N:C}$ (mol N mol C^{-1}) is the N/C ratio of biomass. From (S12) and (S6) under the steady state:

$$V_{NH4+} = DQ_C Y_{Bio}^{N:C} \tag{S13}$$

Finally, by plugging (S13) into (S11), we obtain a further simplified from of X_{NH4+}:

$$X_{NH4+} = \frac{[NH_4^+]_{IN}}{Q_C Y_{Bio}^{N:C}} \tag{S14}$$

Nitrogen fixation case. Since the laboratory study found that the steady state ammonium concentration in the culture was always below detectability [2], we assume that all the incoming ammonium is used for biosynthesis, even when nitrogen fixation is active. When carbohydrate is supplied in sufficient excess, ammonium-based growth does not consume all of it and the excess is used to perform respiratory protection of nitrogenase and dinitrogen-based biosynthesis. In other words, in the nitrogen fixing phase, the cell combines nitrogen fixation and ammonium uptake (if ammonium is present). We define f_{N2} as the fraction of total nitrogen synthesized provided by nitrogen fixation (e.g. when $f_{N2} = 0$ all biosynthesis is based on ammonium uptake). Nitrogen fixation reduces the rate of ammonium uptake required to maintain the associated total biomass production, λ_S:

$$V_{NH4+} = \lambda_S Y_{Bio}^{N:C} \left(1 - f_{N2}\right) \quad \text{(S15)}$$

With (S15) and (S6), (S11) can be modified:

$$X_{N2}^{NH4+} = \frac{[NH_4^+]_{IN}}{Q_C Y_{Bio}^{N:C} \left(1 - f_{N2}\right)} \quad \text{(S16)}$$

By comparing (S16) to (S14), we can see that (S14) occurs when $f_{N2} = 0$ in (S16) and as the ratio of nitrogen fixation f_{N2} increases, cellular density X_{N2}^{NH4+} increases.

Assuming that all of the carbohydrate is consumed places a constraint on the nitrogen fixation rate, effectively the nitrogen fixing cells are carbon limited. Computing cell density from the carbohydrate limited perspective based on equation (S8) but using the whole cell growth efficiency for nitrogen fixation, E_{N2}, instead of E_{CH}:

$$X_{N2}^{CH} = \frac{[CH]_{IN}}{Q_C \left(1 + E_{N2}\right)} \quad \text{(S17)}$$

E_{N2} is calculated based on reaction for N_2 based biosynthesis and respiration based on O_2 concentration (see the section "Determining E_{N2}"). The fraction of nitrogen supplied by nitrogen fixation, f_{N2} is that for

which $X_{N2}^{CH} = X_{N2}^{NH4+}$ which maximizes the cell density, depleting both carbohydrate and ammonium in the culture.

Determining the metabolic regime

The model resolves three possible regimes of cellular metabolism; carbohydrate limited growth (low C/N), ammonium limited growth (moderate C/N), and ammonium-uptake-nitrogen-fixation combined growth (high C/N). We select the optimal regime as that which sustains maximal biomass at steady state under a given set of conditions (i.e. ambient oxygen concentration, the incoming medium concentrations of ammonium and sucrose, and the dilution rate). We first determine whether nitrogen fixation is viable: it can only occur when the intracellular oxygen concentration can be reduced to a critical level (here effectively 0). We evaluate the lowest possible oxygen concentration in vital cells $[O_2]_{Cpot}$ (mol O_2 m^{-3}) based on the carbohydrate availability. When $[O_2]_{Cpot} \leq [O_2]_{cri}$, nitrogen fixation can occur. However, if the exclusive consumption of ammonium leads to a higher population (thus $X_{N2} < X_{NH4+}$), nitrogen fixation is assumed to be zero, since ammonium is energetically more favorable. When $[O_2]_{Cpot} > [O_2]_{cri}$ nitrogen fixation is not viable and growth is either limited by carbohydrate or ammonium, determined by the lower of X_{CH} (cell m^{-3}) and X_{NH4+} (cell m^{-3}) (from equation (S8) and (S14), respectively); effectively a statement of Liebig's law [4]. In essence, we choose the optimal model metabolic configuration based on the criterion of maximizing cell density under any given environmental conditions.

Computing the rate of nitrogen fixation

The rate of nitrogen fixation per cell N_{fix} (mol N cell^{-1} s^{-1}) is simply estimated from the biomass production and f_{N2} since of all the nitrogen incorporated the ratio of f_{N2} originates from nitrogen fixation:

$$N_{fix} = \lambda_S Y_{Bio}^{N:C} f_{N2} \tag{S18}$$

Under the steady state, with (S6), (S18) becomes

$$N_{fix} = DQ_C Y_{Bio}^{N:C} f_{N2} \tag{S19}$$

To obtain the rate of nitrogen fixation per volume culture N_{fix}^{V} (mol N m^{-3}), we multiply (S19) by X_{N_2} :

$$N_{fix}^{V} = DQ_C Y_{Bio}^{N:C} f_{N2} X_{N2} \tag{S20}$$

Computing biomass and protein concentrations

As in [1], we use the biomass formula of $C_6H_{10.8}O_{2.9}N_{1.5}$ [5,3] and dry weight ratio of 1.32 : 1 [3] for the ratio of biomass to protein $Y^{Pro:Bio}$. The biomass concentration in the culture [Bio] (g m^{-3}) is proportional to the population density:

$$[Bio] = XQ_C Y^{Bio:C} \tag{S21}$$

where $Y^{Bio:C}$ is biomass weight to biomass C ratio (g biomass mol C^{-1}) based on the chemical formula $C_6H_{10.8}O_{2.9}N_{1.5}$. Once we obtain [Bio], we simply multiply by $Y^{Pro:Bio}$ (=1.32) to obtain protein concentration [Pro] (g m^{-3}):

$$[Pro] = [\mathrm{Bio}] Y^{Pro:Bio} \tag{S22}$$

Computing the rate of respiration

Averaged respiration rate *Res* (mol O_2 cell^{-1} s^{-1}) is computed differently for different regimes.

Carbohydrate limited case. When carbohydrate is limiting, the respiration rate is based on the energetic demand; thus proportional to biomass production:

$$Res_{CH} = \lambda_S Y_{NH4+}^{O2:Bio} \tag{S23}$$

where Res_{CH} (mol O_2 cell^{-1} s^{-1}) is the averaged respiration rate under carbohydrate limited environment, $Y_{NH4+}^{O2:Bio}$ is the ratio of O_2 consumption to biomass production with ammonium as nitrogen source (see the section "Obtaining stoichiometric parameters" for obtaining $Y_{NH4+}^{O2:Bio}$). Under the steady state, (S6) applies and (S23) becomes

$$Res_{CH} = DQ_C Y_{NH4+}^{O2:Bio} \tag{S24}$$

Ammonium limited case. When ammonium is limited, excess carbon is available and assumed used for respiration to deplete oxygen and enable nitrogen fixation. From (S1), we replace X_{CH} with X_{NH4+} and assume steady state where $[CH]$ becomes small:

$$V_{CH} = \frac{D[CH]_{IN}}{X_{NH4+}} \tag{S25}$$

A fraction of carbohydrate uptake, V_{CH}, is synthesized into new biomass and subtracting the rate of carbohydrate consumption for biomass production C_{CH}^{Bio} (mol C cell^{-1} s^{-1}) from the carbon uptake, the respiratory carbohydrate consumption C_{CH}^{Res} (mol C cell^{-1} s^{-1}) is represented as follows:

$$C_{CH}^{Res} = V_{CH} - C_{CH}^{Bio} \tag{S26}$$

C_{CH}^{Bio} is proportionally related to λ_S with $Y_{Bio-NH4+}^{Con:Pro}$ including all carbohydrate loss for balancing electron, which includes carbohydrate becoming biomass and CO_2:

$$C_{CH}^{Bio} = \lambda_S Y_{Bio-NH4+}^{Con:Pro} \tag{S27}$$

See "Obtaining stoichiometric parameters" to evaluate $Y_{Bio-NH4+}^{Con:Pro}$. Under the steady state (S6) can be used and relate C_{CH}^{Bio} to dilution rate:

$$C_{CH}^{Bio} = DQ_C Y_{Bio-NH4+}^{Con:Pro} \tag{S28}$$

Once we obtain C_{CH}^{Res}, we stoichiometrically relate it to the respiratory oxygen consumption Res_{NH4+} (mol O_2 cell^{-1} s^{-1}):

$$Res_{NH4+} = C_{CH}^{Res} Y_{Res}^{O2:CH} \tag{S29}$$

where $Y_{Res}^{O2:CH}$ (mol O_2 mol C^{-1}) is the ratio of O_2 consumption to carbohydrate consumption in respiratory reaction (see "Obtaining stoichiometric parameters" for $Y_{Res}^{O2:CH}$). By rearranging (S29) with (S25), (S26) and (S28) we relate Res_{NH4+} to dilution rate *D*:

$$Res_{NH4+} = D\left(\frac{[CH]_{IN}}{X_{NH4+}} - Q_C Y_{Bio-NH4+}^{Con:Pro}\right) Y_{Res}^{O2:CH} \tag{S30}$$

Nitrogen fixing case. To obtain the rate of respiration, we compute respiratory demand for two different ways: one based on energetic balance to support cellular metabolism and the other based on O_2 balance to minimize intracellular O_2. Then we take the higher value to fulfill both respiratory demands.

Respiratory demand for energy. To obtain the respiration based on the energy balance Res_{N2fix}^{Energy}, we assume that the energetic demand proportionally increase with the biomass production.

$$Res_{N2fix}^{Energy} = \lambda_S Y_{N2fix}^{O2:Bio} \tag{S31}$$

Where $Y_{N2fix}^{O2:Bio}$ is the ratio of O_2 production to biomass production based on [1,6] (detail in "Obtaining stoichiometric parameters"). Under the steady state, using (S6), we obtain the relation between *D* and Res_{N2fix}^{Energy}:

$$Res_{N2fix}^{Energy} = DQ_C Y_{N2fix}^{O2:Bio} \tag{S32}$$

In the chemostat culture, not all the cells are active; the vitality ratio tend to decrease as the dilution approaches zero [7]. To include this effect we apply vitality ratio P_V and compute the energetic respiratory demand for vital cells $R_{N2fix-vital}^{Energy}$ (mol O_2 cell^{-1} s^{-1}):

$$R_{N2fix-vital}^{Energy} = \frac{DQ_C Y_{N2fix}^{O2:Bio}}{P_V} \tag{S33}$$

where P_V is approximated by

$$P_V = A_V \ln\left(D\right) + B_V \tag{S34}$$

where A_V and B_V are constant.

Respiratory demand for O_2 scavenging. In order to compute the respiratory demand for O_2 scavenging, we consider a passive uptake of O_2 based on diffusion V_{O2} (mol O_2 cell^{-1} s^{-1}):

$$V_{O2} = 4\pi r \kappa_{O2}\left([O_2] - [O_2]_C\right) \tag{S35}$$

where *r* (m) is the radius of cellular cytoplasm assuming a sphere, κ_{O2} (m^2 s^{-1}) is effective diffusion constant of O_2, $[O_2]$ (mol O_2 m^{-3}) is O_2 concentration in the environment and $[O_2]_C$ (mol O_2 m^{-3}) is that in the cytoplasm. We have adapted an equation for O_2 uptake in a spherical cells with reduced diffusivity for cell membrane layers [8]:

$$\kappa_{O2} = \kappa_{O2}^0 \frac{\varepsilon_m \left(r + L_g\right)}{\varepsilon_m r + L_g} \tag{S36}$$

where κ_{O2}^{0} (m^2 s^{-1}) is diffusion constant of O_2 in water, ε_m is the diffusivity of cell membrane layers relative to water, and L_g (m) is the thickness of the cell membrane layers (see "Model parameterization"). For the vital cells fixing nitrogen, we assume $[O_2]_C$ ~ 0, leading to

$$V_{O2} = 4\pi r \kappa_{O2}[O_2] \tag{S37}$$

Under the steady state, this O_2 uptake must be balance with the respiration, thus

$$Res_{N2\,fix-vital}^{O2} = 4\pi r \kappa_{O2}[O_2] \tag{S38}$$

Obtaining the averaged respiration. Once we obtain $Res_{N2\,fix-vital}^{Energy}$ and $Res_{N2\,fix-vital}^{O2}$, we take the higher value and define it as respiration for the vital cells $Res_{N2\,fix-vital}$. Given respiration for non-vital cell is small [7], the averaged respiration for N_2 fixing case Res_{N2} (mol O_2 cell$_{-1}$ s$_{-1}$) is simply a product of $Res_{N2\,fix-vital}$ and P_V:

$$Res_{N2\,fix} = Res_{N2\,fix-vital} P_V \tag{S39}$$

Determining E_{N2}

E_{N2} is the ratio of CO_2 production P_{CO2} (mol C cell^{-1} s^{-1}) to biomass production when nitrogen fixation occurs:

$$E_{N2} = \frac{P_{CO2}}{\lambda_S} \tag{S40}$$

P_{CO2} has two sources; CO_2 production associated with biomass production with nitrogen fixation P_{CO2}^{Bio} and that from respiration P_{CO2}^{Res}.

$$P_{CO2} = P_{CO2}^{Bio} + P_{CO2}^{Res} \tag{S41}$$

Here P_{CO2}^{Bio} is proportional to biomass production λ_S:

$$P_{CO2}^{Bio} = \lambda_S Y_{Syn-N2}^{CO2:Bio} \tag{S42}$$

where $Y_{Syn-N2}^{CO2:Bio}$ is estimated from the biosynthetic reaction reflecting f_{N2} (see Obtaining stoichiometric parameters). P_{CO2}^{Res} is proportional to the respiration rate $Res_{N2\,fix}$:

$$P_{CO2}^{Bio} = \frac{Res_{N2\,fix}}{Y_{Res}^{O2:CH}} \tag{S43}$$

With (S41)~(S43), E_{N2} in (S40) is expanded as follows:

$$E_{N2} = Y_{Syn-N2}^{CO2:Bio} + \frac{Res_{N2\,fix}}{\lambda_S Y_{Res}^{O2:CH}} \tag{S44}$$

and under the steady state with (S6):

$$E_{N2} = Y_{Syn-N2}^{CO2:Bio} + \frac{Res_{N2\,fix}}{DQ_C Y_{Res}^{O2:CH}} \tag{S45}$$

Computation of [O_2]$_{Cpot}$

When O_2 uptake is maximum V_{O2}^{max} (mol O_2 cell^{-1} s^{-1}), intracellular O_2 concentration becomes minimum (thus $[O_2]_{Cpot}$). From (S35):

$$V_{O2}^{max} = 4\pi r \kappa_{O2}\left([O_2]-[O_2]_{Cpot}\right) \tag{S46}$$

and we solve this equation for $[O_2]_{Cpot}$:

$$[O_2]_{Cpot} = [O_2] - \frac{V_{O2}^{max}}{4\pi r \kappa_{O2}} \tag{S47}$$

Under the steady state, V_{O2}^{max} is balanced by maximum respiration rate for the vital cells which equals Res_{NH4+}/P_V. Thus, from (S47) and (S30):

$$[O_2]_{Cpot} = [O_2] - \frac{DY_{Res}^{O2:CH}}{4\pi r \kappa_{O2} P_V}\left(\frac{[CH]_{IN}}{X_{NH4+}} - Q_C Y_{Bio-NH4+}^{Con:Pro}\right) \quad \text{(S48)}$$

Obtaining stoichiometric parameters

To obtain stoichiometric parameters, we refer to the whole cell reaction based on energy, electron and mass balance following [1,6]. We use four half reactions (Table S1); R_a, Respiratory electron acceptance; R_c^{N2}, biomass synthesis with N_2 fixation as nitrogen source; R_c^{NH4+}, biomass synthesis with ammonium uptake as nitrogen source; R_d, carbohydrate decomposition providing electron. We combine them to balance electron and energy and to satisfy f_{N2}.

S1 Table. Fundamental reactions for stoichiometric parameter estimation.

R_a	$\frac{1}{4}O_2 + H^+ + e^- \rightarrow \frac{1}{2}H_2O$
R_c^{N2}	$\left(\frac{n-c}{d}\right)CO_2 + \left(\frac{c}{d}\right)HCO_3^- + \left(\frac{c}{2d}\right)N_2 + \left(\frac{c+d}{d}\right)H^+ + e^- \rightarrow$ $\left(\frac{1}{d}\right)C_nH_aO_bN_c + \left(\frac{2n-b+c}{d}\right)H_2O + \left(\frac{c}{2d}\right)H_2$ where, $d = 4n + a - 2b + c$
R_c^{NH4+}	$\frac{n-c}{d-4c}CO_2 + \frac{c}{d-4c}NH_4^+ + \frac{c}{d-4c}HCO_3^- + H^+ + e^- \rightarrow$ $\frac{1}{d-4c}C_nH_aO_bN_c + \frac{2n-b+c}{d-4c}H_2O$
R_d	$\frac{1}{24}C_6H_{12}O_6 + \frac{1}{4}H_2O \rightarrow \frac{1}{4}CO_2 + H^+ + e^-$

First, we obtain respiratory reaction R_{Res} by simply balancing electron with R_d and R_a:

$$R_{Res} = R_a + R_d \quad \text{(S49)}$$

In a similar way, biomass synthesis with nitrogen fixation R_{Syn}^{N2} is

$$R_{Syn}^{N2} = R_a + R_c^{N2} \quad \text{(S50)}$$

and that with ammonium R_{Syn}^{NH4+} is

$$R_{Syn}^{NH4+} = R_a + R_c^{NH4+} \tag{S51}$$

R_{Syn}^{N2} and R_{Syn}^{N2} consume energy provided by R_{Res}. To balance the energy budget, we combine the reactions to balance energy, leading to the whole cell reaction with all the nitrogen from nitrogen fixation:

$$R_{Cell}^{N2} = f_{Syn}^{N2} R_{Syn}^{N2} + \left(1 - f_{Syn}^{N2}\right) R_{Res} \tag{S52}$$

and that with ammonium uptake as sole nitrogen source:

$$R_{Cell}^{NH4+} = f_{Syn}^{NH4+} R_{Syn}^{NH4+} + \left(1 - f_{Syn}^{NH4+}\right) R_{Res} \tag{S53}$$

where f_{Syn}^{N2} and f_{Syn}^{NH4+} are the ratio of biosynthesis reactions obtained from energy balance (see "Model parameterization"). Finally, we combine R_{Cell}^{N2} and R_{Cell}^{NH4+} with an appropriate ratio to represent the population where f_{N2} of biomass N originates from N_2 fixation:

$$R_{Cell} = f_{Rcell}^{N2} R_{Cell}^{N2} + \left(1 - f_{Rcell}^{N2}\right) R_{Cell}^{NH4+} \tag{S54}$$

where

$$f_{Rcell}^{N2} = \frac{f_{N2} f_{Syn}^{NH4+} d}{f_{N2} f_{Syn}^{NH4+} d + f_{Syn}^{N2}\left(1 - f_{N2}\right)\left(d - 4c\right)} \tag{S55}$$

where c and d (=4n+a-2b+c) are based on biomass stoichiometry $C_nH_aO_bN_c$ (see S1 Table); here $C_6H_{10.8}O_{2.9}N_{1.5}$ is used [5,3]. Once we obtain these reactions, we refer to R_{Cell} for $Y_{N2\,fix}^{O2:Bio}$, R_{Cell}^{NH4+} for E_{CH} and $Y_{NH4+}^{O2:Bio}$, R_{Syn}^{NH4+} for $Y_{Bio-NH4+}^{Con:Pro}$, R_{Syn}^{N2} for $Y_{Syn-N2}^{CO2:Bio}$ and R_{Res} for $Y_{Res}^{O2:CH}$.

Computing fate of carbohydrate

Carbohydrate limited case. Under carbohydrate limited case, carbohydrate used for biomass production is obtained from (S28). The respiration for biomass production $C_{CH}^{Res-bio}$ (mol C cell^{-1} s^{-1}) is obtained from converting Res_{CH} (S24) into carbon:

$$C_{CH}^{Res-bio} = \frac{Res_{CH}}{Y_{Res}^{O2:CH}} \quad \text{(S56)}$$

Ammonium limited case. When ammonium is limited, carbohydrate used for biomass production and respiration for biomass production are obtained in the same way as carbohydrate limited case. The extra respiration rate C_{CH}^{Res-ex} (mol C cell^{-1} s^{-1}) is the difference between total respiration and respiration for biomass production:

$$C_{CH}^{Res-ex} = C_{CH}^{Res} - C_{CH}^{Res-bio} \quad \text{(S57)}$$

Nitrogen fixation case: Under the steady state, with (S42) and (S6) the carbohydrate for biomass production including nitrogen fixation C_{CH}^{Bio-N2} (mol C cell^{-1} s^{-1}) is computed as follows:

$$C_{CH}^{Bio-N2} = \lambda_S + P_{CO2}^{Bio} = DQ_C\left(1 + Y_{Syn-N2}^{CO2:Bio}\right) \quad \text{(S58)}$$

As in (S32) respiration for biomass production including nitrogen fixation $C_{CH}^{Res-bio-N2}$ is

$$C_{CH}^{Res-bio-N2} = \frac{Res_{N2\,fix}^{Energy}}{Y_{Res}^{O2:CH}} \quad \text{(S59)}$$

By subtracting this respiration from the total respiration, we obtain the extra respiration:

$$C_{CH}^{Res-ex} = \frac{Res_{N2\,fix} - Res_{N2\,fix}^{Energy}}{Y_{Res}^{O2:CH}} \quad \text{(S60)}$$

C_{CH}^{Bio-N2} can be stoichiometrically further partitioned into biomass production C_{CH}^{Bio} and nitrogen fixation C_{CH}^{N2fix} [1]:

$$C_{CH}^{Bio} = \frac{d-4c}{d} C_{CH}^{Bio-N2} \tag{S61}$$

and

$$C_{CH}^{N2fix} = \frac{4c}{d} C_{CH}^{Bio-N2} \tag{S62}$$

$C_{CH}^{Res-bio-N2}$ is also partitioned into respiration for biomass production $C_{CH}^{Res-bio}$ and that for nitrogen fixation $C_{CH}^{Res-N2fix}$ based on the energy demands:

$$C_{CH}^{Res-bio} = \frac{(d-4c)\Delta G_s^l}{(d-4c)\Delta G_s^l + 4c\Delta G_n} C_{CH}^{Res-bio-N2} \tag{S63}$$

and

$$C_{CH}^{Res-N2fix} = \frac{4c\Delta G_n}{(d-4c)\Delta G_s^l + 4c\Delta G_n} C_{CH}^{Res-bio-N2} \tag{S64}$$

where ΔG_s^l and ΔG_n are energetic demands per electron for biosynthesis and nitrogen fixation respectively. Detail in these energy demands are described in the supplementary material of [1].

Model parameterization

Cell size is observed to increase with O_2 [9]. We have obtained a linear regression of the width W (m) and length L (m) of the cell and estimated $r + L_g$ as follows:

$$r + L_g = \frac{L/2 + W}{3} \tag{S65}$$

We assume that L_g increases proportionally to increasing r, given that the membrane surface area and number of vesicles increases with the cell radius [9], indicating increased thickness of the cell membrane

layers with radius. We applied the ratio of 29:1 for r : L_g estimated from the microscopic picture (FIG. 2 of [10]). For estimating κ_{O2}^{0} at 30˚C (the temperature in which the laboratory experiment was conducted), we have used κ_{O2}^{0} at 20˚C (= 2.12×10^{-9} [11]) and Walden's rule [12] based on temperature dependent viscosity of water [13]. For ε_m , we use 7.9×10^{-4} [1]. For vitality parameters A_V and B_V, we use 0.185 and 2.75 respectively based on the vitality curve for *Klebsiella* [7]. f_{Syn}^{N2} and f_{Syn}^{NH4+} are computed to satisfy the energy balance with energy transfer efficiency ε of 0.22 for f_{Syn}^{N2} [1] and 0.54 for f_{Syn}^{NH4+} (estimated from [14]), leading to f_{Syn}^{N2} = 0.224 and f_{Syn}^{NH4+} = 0.653 (detail of balancing energy is in [1] for f_{Syn}^{N2} and [6] for f_{Syn}^{NH4+}). We use ΔG_s^l and ΔG_n of 91.4 and 90.9 respectively as computed with ε of 0.22 [1]. For the cellular carbon Q_C, we have applied 0.22 g of C cm^{-3} and the cellular volume V (m^3) assuming complete sphere:

$$V = \frac{4}{3}\pi\left(r + L_g\right)^3 \tag{S66}$$

S2 Table. Nomenclature of the used symbols in the supplementary material. The symbols are roughly in the order of appearance.

Symbol	Definition	Unit
C/N	Sucrose to ammonium ratio	mol sucrose mol N^{-1}
$[CH]$	Carbohydrate concentration in the chemostat vessel	mol C m^{-3}
t	Time	s
D	Dilution rate (average growth rate)	s^{-1}
$[CH]_{IN}$	Carbohydrate concentration in the incoming medium	mol C m^{-3}
V_{CH}	Averaged carbohydrate uptake rate per cell	mol C $cell^{-1}$ s^{-1}
X_{CH}	Number density of the cell under carbohydrate limitation	cell m^{-3}
CH_C	Intermediate cellular carbohydrate	mol C $cell^{-1}$
λ_S	Averaged biomass production rate per cell	mol C $cell^{-1}$ s^{-1}
E_{CH}	The ratio of CO_2 production to biomass production under carbohydrate limitation	mol C mol C^{-1}
Q_C	Cellular C quota	mol C $cell^{-1}$

μ	Cellular growth rate μ	s^{-1}
$[NH_4^+]_{IN}$	Ammonium concentration in the incoming medium	mol N m^{-3}
$[NH_4^+]$	Ammonium concentration in the chemostat vessel	mol N m^{-3}
X_{NH4+}	Number density of cells under ammonium limitation	cell m^{-3}
V_{NH4+}	Averaged ammonium uptake rate	mol N $cell^{-1}$ s^{-1}
$NH_{4\,Cell}^{+}$	Cellular ammonium quota	mol N $cell^{-1}$
$Y_{Bio}^{N:C}$	N/C ratio of biomass	mol N mol C^{-1}
X_{N2}^{NH4+}	Number density of cells when nitrogen fixation occurs computed based on ammonium limitation	cell m^{-3}
X_{N2}^{CH}	Number density of cells when nitrogen fixation occurs computed based on carbohydrate limitation	cell m^{-3}
X_{N_2}	Number density of cells when nitrogen fixation occurs	cell m^{-3}
f_{N2}	The ratio of nitrogen from nitrogen fixation to all the nitrogen sources	mol N mol N^{-1}
E_{N2}	The ratio of CO_2 production to biomass production when nitrogen fixation occurs	mol C mol C^{-1}
$[O_2]_{Cpot}$	The lowest possible oxygen concentration in vital cells	mol O_2 m^{-3}
$[O_2]_{cri}$	Critical intracellular oxygen concentration for nitrogen fixation	mol O_2 m^{-3}
N_{fix}	The averaged rate of nitrogen fixation per cell	mol N $cell^{-1}$ s^{-1}
N_{fix}^{V}	The rate of nitrogen fixation per volume	mol N $cell^{-1}$ s^{-1}
$Y^{Pro:Bio}$	Dry weight ratio of protein to biomass	g g^{-1}
$Y^{Bio:C}$	Biomass weight to biomass C ratio	g biomass mol C^{-1}
$[Bio]$	Biomass concentration in the culture	g m^{-3}
$[Pro]$	Protein concentration in the culture	g m^{-3}
Res	Averaged respiration rate	mol O_2 $cell^{-1}$ s^{-1}
Res_{CH}	Averaged respiration rate under carbohydrate limitation	mol O_2 $cell^{-1}$ s^{-1}
$Y_{NH4+}^{O2:Bio}$	The ratio of O_2 consumption to biomass production under ammonium limitation	mol O_2 mol C^{-1}
C_{CH}^{Bio}	The averaged rate of carbohydrate consumption for biomass production	mol C $cell^{-1}$ s^{-1}
$Y_{Bio-NH4+}^{Con:Pro}$	The ratio of C consumption for biomass production to biomass production under ammonium limitation	mol C mol C^{-1}
Res_{NH4+}	The averaged respiration rate under ammonium limitation	mol O_2 $cell^{-1}$ s^{-1}

$Y_{Res}^{O2:CH}$	The ratio of O_2 consumption to carbohydrate consumption in respiratory reaction	mol O_2 mol C^{-1}
$Res_{N2\,fix}^{Energy}$	The averaged rate of energetically balanced respiration when nitrogen fixation occurs	mol O_2 cell^{-1} s^{-1}
$Y_{N2\,fix}^{O2:Bio}$	The ratio of O_2 consumption to biomass production when nitrogen fixation occurs	mol O_2 mol C^{-1}
$R_{N2\,fix-vital}^{Energy}$	The energetic respiratory demand for vital cells	mol O_2 cell^{-1} s^{-1}
P_V	Vitality ratio of the cells	cell cell^{-1}
A_V	Coefficient for vitality ratio for the variable term	cell cell^{-1}
B_V	Coefficient for vitality ratio for the constant term	cell cell^{-1}
V_{O2}	O_2 uptake rate	mol O_2 cell^{-1} s^{-1}
r	Radius of cellular cytoplasm	m
κ_{O2}	Effective diffusion constant of O_2	m^2 s^{-1}
$[O_2]$	O_2 concentration in the environment	mol O_2 m^{-3}
$[O_2]_C$	Cytoplasmic O_2 concentration	mol O_2 m^{-3}
κ_{O2}^0	Diffusion constant of O_2 in water	m^2 s^{-1}
ε_m	diffusivity of cell membrane layers relative to water	m^2 s^{-1} (m^2 s^{-1})$^{-1}$
L_g	Thickness of the cell membrane layers	m
$Res_{N2\,fix-vital}^{O2}$	Respiratory demand for O_2 scavenging	mol O_2 cell^{-1} s^{-1}
$Res_{N2\,fix-vital}$	Respiration rate by the vital cells	mol O_2 cell^{-1} s^{-1}
$Res_{N2\,fixl}$	Averaged respiration rate	mol O_2 cell^{-1} s^{-1}
P_{CO2}	CO_2 production rate	mol C cell^{-1} s^{-1}
P_{CO2}^{Bio}	CO_2 production associated with biomass production	mol C cell^{-1} s^{-1}
P_{CO2}^{Res}	CO_2 production from respiration	mol C cell^{-1} s^{-1}
$Y_{Syn-N2}^{CO2:Bio}$	The ratio of CO_2 production to biomass production in biosynthesis reaction	mol C mol C^{-1}
R_a	Half reaction for respiratory electron acceptance	-
R_c^{N2}	Half reaction for biomass synthesis with nitrogen fixation	-
R_c^{NH4+}	Half reaction for biomass synthesis with nitrogen fixation with ammonium	-
R_d	Half reaction for electron donation from carbohydrate	-
R_{Res}	Respiratory reaction	-
R_{Syn}^{N2}	Biosynthesis reaction with nitrogen fixation	-
R_{Syn}^{NH4+}	Biosynthesis reaction with ammonium	-

R_{Cell}^{N2}	Whole cell reaction with all the nitrogen from nitrogen fixation	-
f_{Syn}^{N2}	The ratio of R_{Syn}^{N2} under energetically balanced state	-
R_{Cell}^{NH4+}	Whole cell reaction with all the nitrogen from ammonium	-
f_{Syn}^{NH4+}	The ratio of R_{Syn}^{NH4+} under energetically balanced state	-
R_{Cell}	Whole cell reaction with nitrogen fixation and ammonium uptake combined with the ratio of f_{N2} : 1- f_{N2}	-
f_{Rcell}^{N2}	The ratio of R_{Cell}^{N2} when nitrogen fixation and ammonium uptake are combined with the ratio of f_{N2} : 1- f_{N2}	-
$C_{CH}^{Res-bio}$	The rate of carbohydrate consumption for respiration for biosynthesis	mol C cell^{-1} s^{-1}
C_{CH}^{Res-ex}	The rate of carbohydrate consumption for extra respiration	mol C cell^{-1} s^{-1}
C_{CH}^{Bio-N2}	The rate of carbohydrate consumption for biosynthesis and nitrogen fixation	mol C cell^{-1} s^{-1}
$C_{CH}^{Res-bio-N2}$	The rate of carbohydrate consumption for respiration supporting biosynthesis and nitrogen fixation	mol C cell^{-1} s^{-1}
C_{CH}^{N2fix}	The rate of carbohydrate consumption for providing electron for nitrogen fixation	mol C cell^{-1} s^{-1}
$C_{CH}^{Res-N2fix}$	The rate of carbohydrate consumption for respiration for nitrogen fixation	mol C cell^{-1} s^{-1}
ΔG_s^l	Free energy demand for biosynthesis with ammonium as a nitrogen source	kJ/e^-
ΔG_n	Free energy demand for nitrogen fixation and electron donation	kJ/e^-
L	Cell length based on observation	m
W	Cell width based on observation	m
V	Cell volume	m^3

References

1. Inomura K, Bragg J, Follows MJ. A quantitative analysis of the direct and indirect costs of nitrogen fixation: a model based on Azotobacter vinelandii. ISME J. 2017;11: 165–175. doi:10.1038/ismej.2016.97

2. Bühler T, Sann R, Monter U, Dingier C, Kuhla J, Oelze J. Control of dinitrogen fixation in

ammonium-assimilating cultures of *Azotobacter vinelandii*. Arch Microbiol. 1987;148: 247–251. doi:https://doi.org/10.1007/BF00414820

3. Bühler T, Monter U, Sann R, Kuhla J, Dingier C, Oelze J. Control of respiration and growth yield in ammonium-assimilating cultures of *Azotobacter vinelandii*. Arch Microbiol. 1987;148: 242–246. doi:https://doi.org/10.1007/BF00414819

4. De Baar HJW. von Liebig's law of the minimum and plankton ecology (1899-1991). Prog Oceanogr. 1994;33: 347–386.

5. van Verseveld, H W. Influence of environmental factors on the efficiency of energy conservation in *Paracoccus denitrificans*. PhD-thesis. 1979;

6. Rittmann BE, McCarty PL. Stoichiometry and bacterial energetics. In: Environmental Biotechnology: Principles and Applications. McGraw-Hill: New York. McGraw-Hill; 2001. pp. 126–164.

7. Postgate JR. The viability of very slow-growing populations: A model for the natural ecosystem. Bull Ecol Res Comm. 1973;17: 287–292.

8. Staal M, Meysman FJR, Stal LJ. Temperature excludes N_2-fixing heterocystous cyanobacteria in the tropical oceans. Nature. 2003;425: 504–507. doi:10.1038/nature02001.1.

9. Post E, Golecki JR, Oelze J. Morphological and ultrastructural variations in *Azotobacter vinelandii* growing in oxygen-controlled continuous culture. Arch Microbiol. 1982;133: 75–82.

10. Oppenheim J, Marcus L. Correlation of ultrastructure in *Azotobacter vinelandii* with nitrogen source for growth. J Bacteriol. 1970;101: 286–291.

11. MacDougall JDB, McCabe M. Diffusion coefficient of oxygen through tissues. Nature. 1967;215:

1173–1174.

12. Fernandez AC, Phillies GDJ. Temperature dependence of the diffusion coefficient of polystyrene latex spheres. Biopolymers. 1983;22: 593–595. doi:10.1002/bip.360220203

13. Kestin J, Sokolov M, Wakeham WA. Viscosity of liquid water in the range -8°C to 150°C. J Phys Chem Ref Data. 1978;7: 941–948.

14. Heijnen JJ, Roels JA. A macroscopic model describing yield and maintenance relationships in aerobic fermentation processes. Biotechnol Bioeng. 1981;23: 739–763.
