# Supplementary material for: A quantitative model of nitrogen fixation in the presence of ammonium
Source: PLoS One. 2018 Nov 29;13(11):e0208282. doi: 10.1371/journal.pone.0208282 (PMC6264846; doi:10.1371/journal.pone.0208282)
Supplement: S2 Text — (PDF) [file pone.0208282.s002.pdf]

Fitness advantage of using fixed nitrogen and dinitrogen

Resource Ratio Theory [1–3] provides a mathematical context for considering the viability of nitrogen fixation relative to less expensive sources of nitrogen in particular in the marine context [4,5]. Broadly speaking, diazotrophs and fixed nitrogen users can co-exist when the latter are N-limited and the former are limited by another resource (e.g. P, Fe). The relative rate of supply of the two limiting resources, and the elemental demands of the organisms, define the regimes where nitrogen fixation can co-exist. Broadly following from, and extending, Dutkiewicz et al. [4], consider the implications of the diazotroph also assimilating fixed nitrogen.

Governing equations for a nitrogen-limited fixed-nitrogen consumer, biomass *B* (in N), and a phosphorus-limited diazotroph, biomass *D* (in N), which also assimilates fixed nitrogen, are:

$$\frac{\partial B}{\partial t} = \mu_B \frac{N}{N + K_N} B - m_B B$$

$$\frac{\partial D}{\partial t} = \mu_D \frac{P}{P + K_P} D - m_D D$$

Here, using Monod kinetics and simple mortality to describe the populations, where *B* and *D* are limited by limited by N and P, respectively. μ_i is the maximum rate of population growth, and m_i is the mortality rate of the *i*th cell type ($i = B$ for non-diazotroph, and $i = D$ for diazotroph). K_N is the half-saturation for consumption of fixed nitrogen (for *B*), and K_P is the half-saturation for consumption of phosphorus (for *D*). *N* and *P* are the concentrations of fixed nitrogen and phosphorus, respectively. Associated governing equations for the fixed-nitrogen, N, and phosphorus, P, sources are

$$\frac{\partial N}{\partial t} = -\mu_B \frac{N}{N + K_N} B - f \mu_D \frac{P}{P + K_P} D + S_N$$

$$\frac{\partial P}{\partial t} = -Y_B^{PN}\mu_B \frac{N}{N+K_N} B - Y_D^{PN}\mu_D \frac{P}{P+K_P} D + S_P$$

Here, f is the fraction of total diazotroph growth which is supported by assimilation of fixed nitrogen (thus $=1-f_{N2}$ of the main text), Y_i^{PN} is the P:N of each cell, S_N and S_P are the rates of supply of fixed nitrogen and phosphorus (respectively) to the system.

Assuming steady state and solving for the respective biomasses of the non-diazotroph and diazotroph, B and D respectively, we find:

$$B = \frac{1}{m_B}\left(S_N - \frac{f(Y_B^{PN}S_N - S_P)}{(Y_D^{PN} - fY_B^{PN})}\right)$$

$$D = \frac{1}{m_D}\left(\frac{(Y_B^{PN}S_N - S_P)}{(Y_D^{PN} - fY_B^{PN})}\right)$$

Compare the case when the diazotrophs do not utilize fixed nitrogen, $f=0$, with the case where they do, $0<f<1$. When the diazotroph uses both nitrogen sources it has a higher steady state biomass at the expense of that of the non-diazotroph. The former has taken some of the latter's limiting resource, increasing its own fitness. This will increase the range of nutrient supply ratios under which the diazotroph is able to co-exist with the non-diazotroph.

References

1. Tilman D. Resource competition between plankton algae: An experimental and theoretical approach. Ecology. 1977;2: 338–348.
2. Tilman D. Resources: A Graphical-Mechanistic Approach to Competition and Predation. Am Nat. 1980;116: 362–393.
3. Tilman D. Resource competition and community structure. Princeton University Press. 1982.
4. Dutkiewicz S, Ward BA, Monteiro F, Follows MJ. Interconnection of nitrogen fixers and

iron in the Pacific Ocean: Theory and numerical simulations. Global Biogeochem Cycles. 2012;26: GB1012, doi:10.1029/2011GB004039. doi:10.1029/2011GB004039

5. Ward BA, Dutkiewicz S, Moore CM, Follows MJ. Iron, phosphorus, and nitrogen supply ratios define the biogeography of nitrogen fixation. Limnol Oceanogr. 2013;58: 2059–2075. doi:10.4319/lo.2013.58.6.2059
