# Supplementary material for: A quantitative model of nitrogen fixation in the presence of ammonium
Source: PLoS One. 2018 Nov 29;13(11):e0208282. doi: 10.1371/journal.pone.0208282 (PMC6264846; doi:10.1371/journal.pone.0208282)
Supplement: S1 Fig — (PDF) [file pone.0208282.s003.pdf]

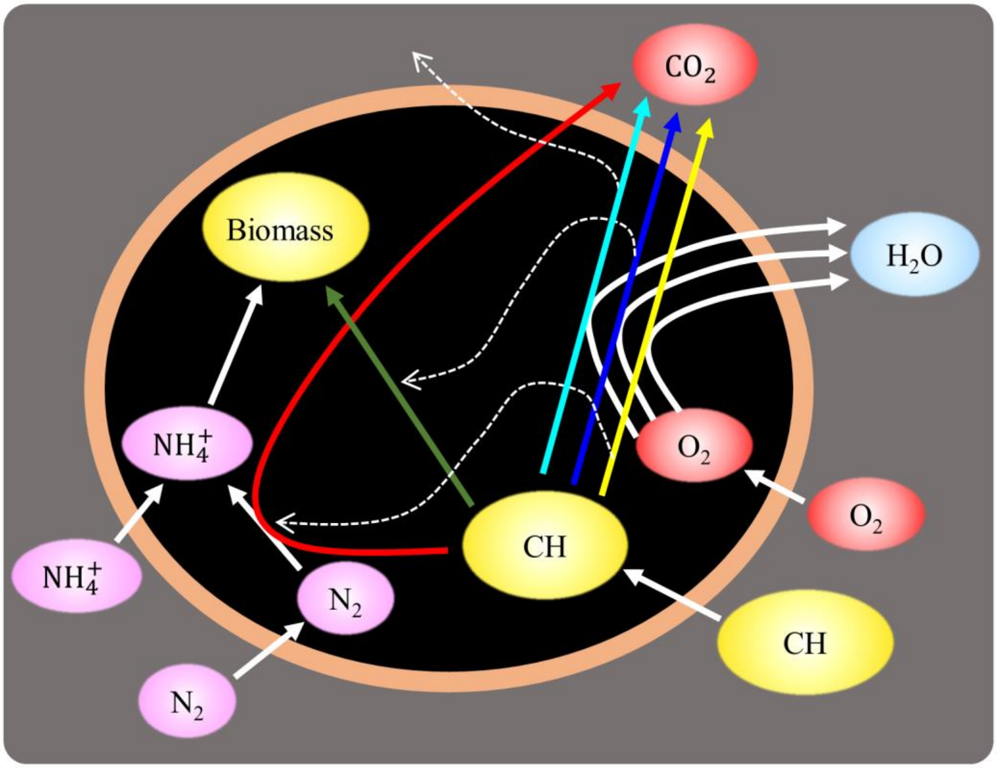

S1 Fig. Schematic of detailed carbon fluxes in a modeled cell. Cyan arrow: carbohydrate (CH) fluxes for excess respiration. Blue arrow: CH used for respiration, which provides energy for biomass production. Yellow arrow: CH used for respiration for providing energy for nitrogen fixation. Red arrow: CH used for electron donation for nitrogen fixation. Green arrow: CH used for biomass production. Solid white arrows are all other chemical fluxes. Dashed white arrows represent energy fluxes. The color scheme of the fluxes (except for white) corresponds to CH fluxes in Fig 4. Black is the intracellular space, peach is the cell membrane layers and gray is the environment.
