# Supplementary material for: A quantitative model of nitrogen fixation in the presence of ammonium
Source: PLoS One. 2018 Nov 29;13(11):e0208282. doi: 10.1371/journal.pone.0208282 (PMC6264846; doi:10.1371/journal.pone.0208282)
Supplement: S2 Fig — (PDF) [file pone.0208282.s004.pdf]

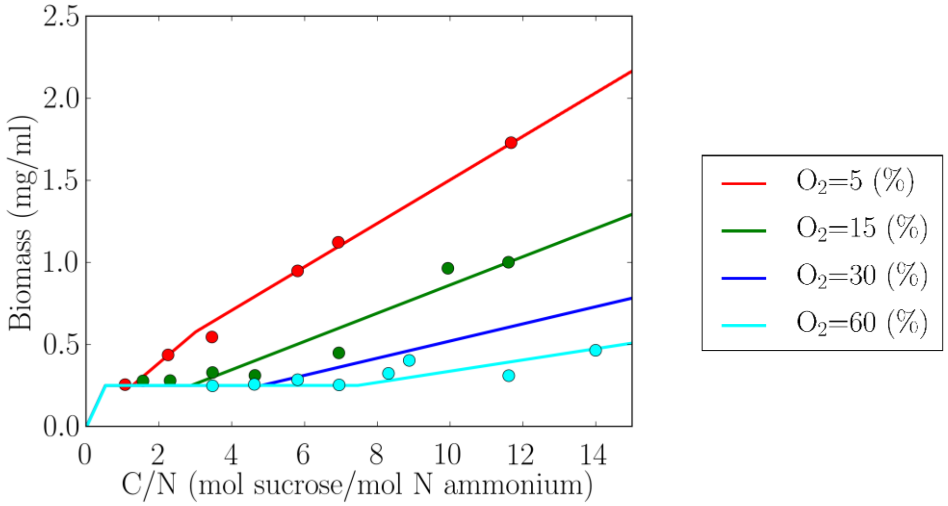

S2 Fig. Simulated concentrations of biomass in continuous cultures of *Azotobacter vinelandii*. The simulations (solid curves) are compared to laboratory data (points) redrawn from [1]. Different colors represent different O_2 concentration in the culture (see the legend in upper right). Here 100% O_2 equals 225 µM thus approximately O_2 saturation under normal air composition at 30 ºC. At lower to medium C/N, model results show same values for various O_2 concentrations. In both the simulation and the laboratory data, the dilution rate was constant (0.15 h^{-1}), and C/N ratio is based on the constant ammonium resource of 2.5 mol m^{-3}.

Reference

1. Bühler T, Monter U, Sann R, Kuhla J, Dingier C, Oelze J. Control of respiration and growth yield in ammonium-assimilating cultures of *Azotobacter vinelandii*. Arch Microbiol. 1987;148: 242–246. doi:https://doi.org/10.1007/BF00414819
