# Supplementary material for: A quantitative model of nitrogen fixation in the presence of ammonium
Source: PLoS One. 2018 Nov 29;13(11):e0208282. doi: 10.1371/journal.pone.0208282 (PMC6264846; doi:10.1371/journal.pone.0208282)
Supplement: S3 Fig — (PDF) [file pone.0208282.s005.pdf]

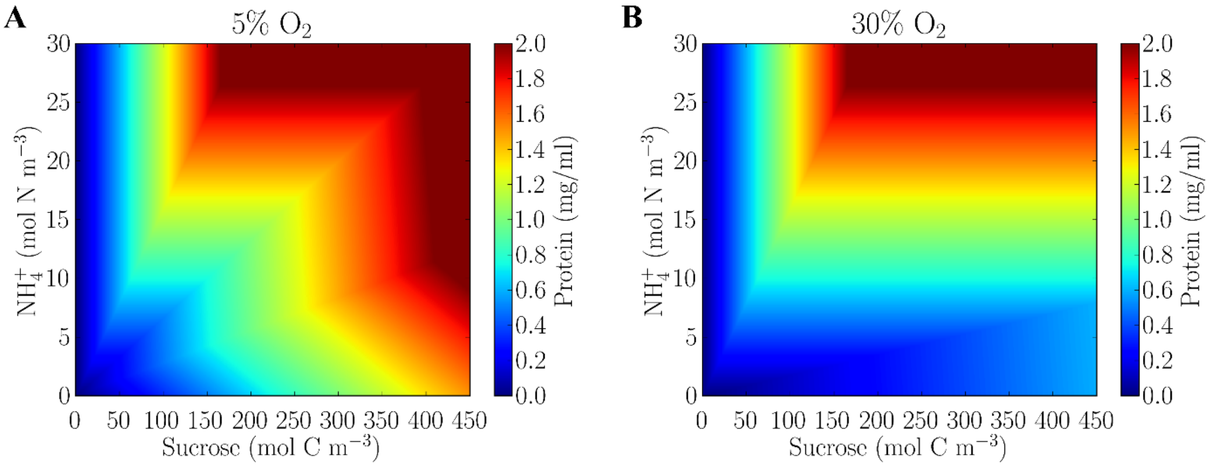

S3 Fig. Cell density proxied by protein concentration for various sucrose and ammonium resources with different oxygen saturations. (A) 5% O_2 and (B) 30% O_2. 100% O_2 equals 225 µM thus approximately O_2 saturation under normal air composition at 30 ºC. Dilution rate is constant at 0.15 h^{-1}.
